# A Network Characteristic That Correlates Environmental and Genetic Robustness

**DOI:** 10.1371/journal.pcbi.1003474

**Published:** 2014-02-13

**Authors:** Zeina Shreif, Vipul Periwal

**Affiliations:** Laboratory of Biological Modeling, National Institutes of Diabetes and Digestive and Kidney Diseases, National Institutes of Health, Bethesda, Maryland, United States of America; University of Chicago, United States of America

## Abstract

As scientific advances in perturbing biological systems and technological advances in data acquisition allow the large-scale quantitative analysis of biological function, the robustness of organisms to both transient environmental stresses and inter-generational genetic changes is a fundamental impediment to the identifiability of mathematical models of these functions. An approach to overcoming this impediment is to reduce the space of possible models to take into account both types of robustness. However, the relationship between the two is still controversial. This work uncovers a network characteristic, transient responsiveness, for a specific function that correlates environmental imperturbability and genetic robustness. We test this characteristic extensively for dynamic networks of ordinary differential equations ranging up to 30 interacting nodes and find that there is a power-law relating environmental imperturbability and genetic robustness that tends to linearity as the number of nodes increases. Using our methods, we refine the classification of known 3-node motifs in terms of their environmental and genetic robustness. We demonstrate our approach by applying it to the chemotaxis signaling network. In particular, we investigate plausible models for the role of CheV protein in biochemical adaptation via a phosphorylation pathway, testing modifications that could improve the robustness of the system to environmental and/or genetic perturbation.

## Author Summary

Advances in the ways that living systems can be perturbed in order to study how they function and sharp reductions in the cost of computer resources have allowed the collection of large amounts of data. The aim of biological system modeling is to analyze this data in order to pin down the precise interactions of molecules that underlie the observed functions. This is made difficult due to two features of biological systems: (1) Living things do not show an appreciable loss of function across large ranges of environmental factors. (2) Their function is inherited from parent to child more or less unchanged in spite of random mutations in genetic sequences. We find that these two features are more correlated in a specific subset of networks and show how to use this observation to find networks in which these two features appear together. Working within this smaller space of networks may make it easier to find suitable underlying models from data.

## Introduction

Biological systems in general show various types and degrees of robustness to environmental changes, meaning that they continue to function even when changes in the environment occur. This imperturbability is often accompanied by robustness to genetic perturbations, meaning that progeny function even though their genotype is not identical to the parent genotype [1]–[4]. Both features play an important role in evolutionary biology. While the former is a direct outcome of selection, the relationship between evolution and genetic robustness is likely to be indirect for low functional mutation rates [5]–[7] since selection acts only on the phenotype of an organism and not its genotype [8].

It has been argued that the ability of an organism to withstand genetic mutations improves its ability to evolve [8]–[11]. However, the rationale for selection for genetic robustness is still controversial [5]–[8], [12]–[14]. A correlation between the evolution of environmental and genetic robustness has been proposed [1], [8], [15], [16] based on examples observed in many biological systems such as in yeast [1], bacterial sncRNAs [2], segment polarity in the fruit-fly [3], bacterial chemotaxis [4], [17]–[24], heat-shock proteins [25], [26], and miRNA stem-loop structures in various species [27] and based on numerical models of evolution under varying fitness conditions [15], [16]. Similarly, it has been shown that metabolic networks evolving under fluctuating environments acquire robustness to the loss of certain genes as well, while those evolving under stable environments do not [28]. However, there is no general mathematical proof for this correlation [8].

In this study, we develop a computational experiment to investigate the plausibility of this hypothesis, that there is a general correlation between environmental and genetic robustness, and provide a quantitative measure of the degree of correlation, if any. In more detail, we shall show that the presence of a specific dynamic network characteristic in networks is associated with a better correlation between genetic and environmental robustness than found in networks where it is absent. Rather than focusing on a particular system in a specific organism, we choose one function of interest: The ability to attain steady state output for constant input. If a network capable of carrying out this function is robust to external environmental perturbations, what is the probability that it is also robust to internal (e.g., genetic) disruption? To be specific, we define environmental robustness of a biological network as the ability to maintain an output in the face of input perturbations. Genetic robustness is defined as the ability of a biochemical system to maintain the same output in the face of genetic mutations represented as rate constant changes in the equations representing it. This representation of a mutation as a jump from one set of parameters to another is a standard assumption [29].

For mathematical convenience, we restrict our discussion to Michaelis-Menten type networks as they are likely to reach a steady state under constant inputs relative to general networks without sigmoidal saturation. Such networks were also used in the analysis of three node biochemically adaptable networks by Ma et al. [30]. The sensitivity of biochemical kinetic models to parameter perturbations has been intensively investigated [29], [31]–[35] as a mathematical model of a biological system should be able to reproduce the function of interest or fit experimental data with a minimal need for parameter fine-tuning [35], [36]. Systems of biochemical adaptation [30], [37]–[40] have been of interest in particular.

Defining a topology to be a graph of interactions independent of parameter values, we test a large number of random *N*-node topologies for networks capable of reaching a steady state both under constant input concentrations and after a persistent step change in these input concentrations. We define a network as a topology with a specific set of parameters. Each network is given a numerical value for its level of robustness to input and parameter perturbations. The level of robustness of the topology is determined by averaging over this value obtained from its corresponding networks. In particular, we differentiate between networks that show a transient response to a step change in input and those that do not. We find that there is a statistically significant model II regression between the level of robustness to input of a topology and its level of robustness to parameter perturbations that has a steeper slope in networks with a transient response. Our results may be relevant to the discussion about the relationship between the need to survive in a constantly changing environment and the evolution of genetic robustness.

There is a large literature on functional motifs that are necessary for a biological system to carry out specific tasks [30], [41]–[49]. Here, we test all possible 3-node topologies to find the particular motifs that are of use in achieving both robustness to input and parameters. Having established the correlation between environmental and genetic robustness, we ask if there are topologies sharing certain sets of motifs/architectures that show stronger correlations than others. Ma et al [30] computationally explored all possible topologies of 3-node Michaelis-Menten enzymatic networks for motifs that can best accomplish biochemical adaptation. Using our results on this correlation between different sets of architectures we refine the list of motifs of biochemical adaptations previously published [30].

Our approach can be used to select/reject plausible/improbable models of a system of interest. We demonstrate this via a comparative study of bacterial chemotaxis signaling systems. Chemotaxis is a process generally used by bacteria to sense changes in their chemical environment [4], [17]–[24]. Chemotactic signaling is a well-studied system, but most of the focus has been on the chemotaxis network of the Escherichia coli (E. coli) bacterium [4], [17]–[20] despite the fact that chemotactic signaling pathways differ between species [21]–[24]. For instance, CheV is a chemotaxis protein found in many bacteria but not in E. coli. In many species, it was shown that CheV, or a variant of it, plays a role in biochemical adaptation during chemotaxis via its phosphorylatable receiver domain [24], [50], [51]. However, the exact mechanism is still not known [24]. Here, we compare the coarse-grained network of E. coli chemotaxis with several others involving CheV phosphorylation. We draw conclusions based on the resultant values of robustness to both input and parameter perturbations and the correlation between them.

In summary, we provide extensive evidence for a mathematical principle stating that, statistically speaking, dynamical systems that are biochemically adaptable are also genetically robust. We apply this knowledge to search for topological categories and subcategories within 3-node networks that show a particularly strong correlation and a linear relationship between their robustness to input and to parameter perturbations, and to shed more light on the chemotactic signaling pathways in bacteria. This method of searching for motifs can be extended to other functions and to bigger networks in order to find motifs that combine more complex functions necessitating larger numbers of nodes.

## Results

**Figure 1:**
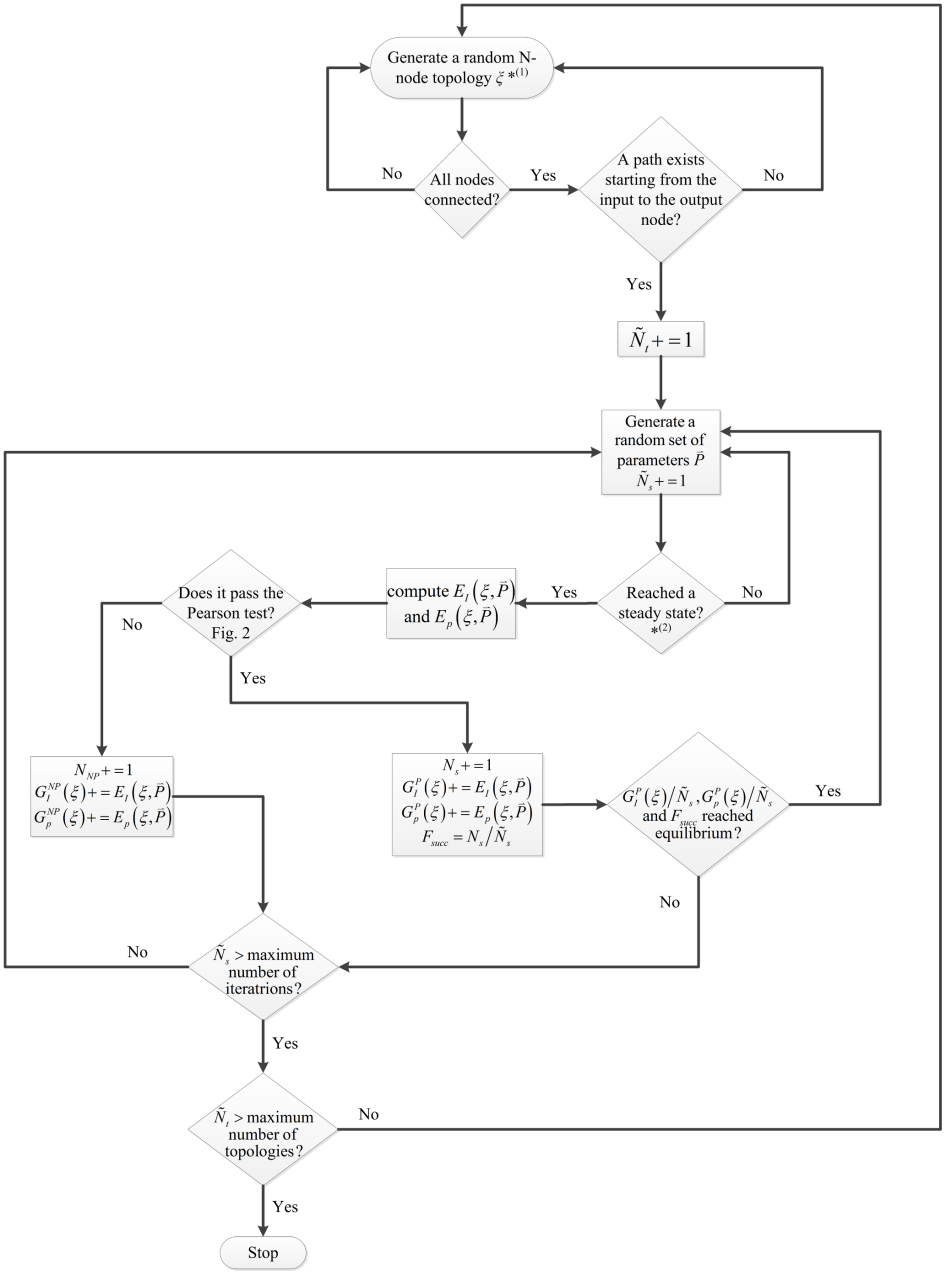
Flowchart for our methodology. 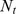
 = the total number of tested random topologies. 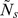 = the total number of trials, i.e., different sets of parameters tested. *^(1)^ for the 3-node networks the topologies are not randomly generated, rather sequentially in order to test all 3^9^ possible combinations. *^(2)^ if the network takes too long to reach equilibrium or the Jacobean matrix 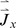 (Eq. A4) is invertible, then the network is rejected.

In the current work, we sample over 50,000 topologies each of 5-node, 10-node, 15-node, and 30-node networks, and over all 3^9^ possible topologies of 3-node networks. For each topology 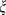, we average over a large number of randomly chosen parameter sets. The parameters are chosen from a uniform distribution within fixed ranges as described in the Methods section. For each network defined by topology 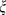 and parameter set 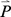
_,_ we compute two values: 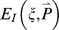 which is a measure of the robustness of the network to a persistent step change in input, and 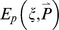 which is a measure of the robustness of the network to perturbations in its set of parameters 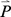. We take the geometric averages of 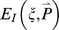 and 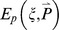 over the whole parameter space as a quantitative evaluation of the robustness of topology 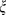 to a step change in input and to parameter perturbations respectively (Fig. 1).

**Figure 2:**
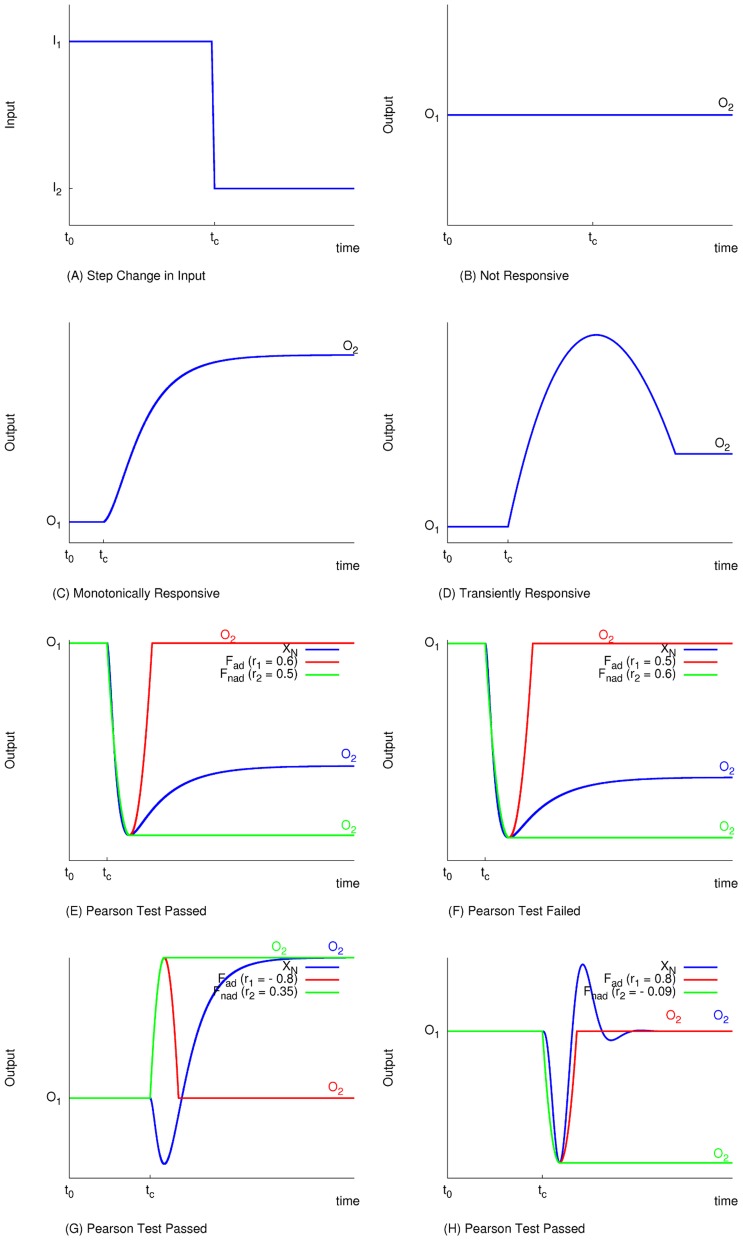
Illustrating the criterion for selecting for transiently responsive networks. The Pearson test is performed to decide whether a network (a particular choice of a set of parameters for a particular topology) shows a transient response to a step change in input or not. The starting point is at steady state under a constant input concentration 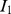. At time 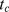 the input concentration is changed from 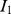 to 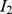 via a step function (A). Consequently the output will either (B) not sense the change and maintain the same steady state 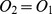, (C) change monotonically to a new steady state 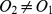, or (D) show a transient response followed by a relaxation to a new steady state 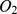 that might or might not be equal to the pre-step change steady state 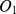. A network passes the test only if it is transiently responsive. (E–H) 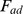 (red) is a perfectly biochemically adaptable function and 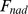 (green) is a monotonically changing function. If The Pearson shape correlation between the computed time course (blue) and 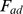, 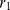, is bigger than that between it and 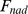, 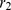, then the test is passed (E, G, H) and the network is termed a Transiently- Responsive (TR) network, otherwise the test is failed (F) and the network is termed Non-Pearson (NP). In (E) and (F), we show two cases where a biased visual inspection would deem both networks looking similar, but in reality they are distinguished by the Pearson test. A biochemically adaptable network is one that is both transiently responsive and returns to a new steady state very close to the pre-step change steady state. For example, (G) is not biochemically adaptable as there is a large difference between the pre-step change steady state and the new one. On the other hand, (H) is perfectly biochemically adaptable as it is both transiently responsive and perfectly robust, i.e. it returns exactly to its pre-step change steady state.

In previous work on biochemical adaptability, it was assumed that networks that quickly respond to input change are better adapted than those with slower response [30], [37]. In this work, we take a qualitative approach to avoid a bias towards larger or faster transients. A biochemically adaptable network is defined as one that is both robust to input perturbations and has a transient response to a persistent step change in input, independent of the magnitude of the transient. A step change in input (Fig. 2A) induces three possible responses from the output dynamics (assuming a steady state can be reached): No response (Fig. 2B), a monotonic response (Fig. 2C), or a transient response (Fig. 2D). Due to possible computational noise in the time-course of the output concentration, we need an objective way to distinguish between a network with a small transient and a non-responsive or monotonically responsive one (e.g., Fig. 2E,F). To this end, we evaluate the Pearson shape correlation between the network's time-course and two model time-courses representing the dynamics of a network characterized by perfect biochemical adaptation (the red time-courses in Fig. 2E–H) and that of a monotonically responsive one (the green time-courses in Fig. 2E–H). Here, the time-course represents the dynamics of the concentration of the output node from one steady state (before the change in input concentration) to a new steady state (after a persistent change in input concentration). In summary, we use the term “transiently-responsive” (TR) for a network that responds to a persistent step change in input and then returns to a new steady state different than the peak response regardless of whether it is also robust (Fig. 2H) or not (Fig. 2G). Any network that does not pass the Pearson test, whether it shows no response or a monotonic one, is termed Non-Pearson (NP). A perfectly biochemically adaptable network is one that is both transiently-responsive and perfectly robust to input perturbations (Fig. 2H).

### Correlation between Robustness to Input and Parameter Perturbations

We define and derive (see *Methods*) two quantitative measures of input and parameter robustness for each topology 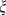: 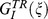, 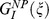, 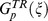, and 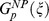. 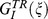 and 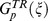 are the values of input and parameter robustness of TR networks (networks that passed the Pearson test) while 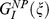 and 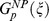 are the values of input and parameter robustness of NP networks (networks that did not pass the Pearson test). A topology 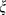 is perfectly robust to input perturbations if 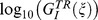 is very small, and similarly 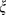 is perfectly robust to parameter perturbations if 
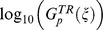
 is very small (i.e., has a very large negative value).

**Figure 3:**
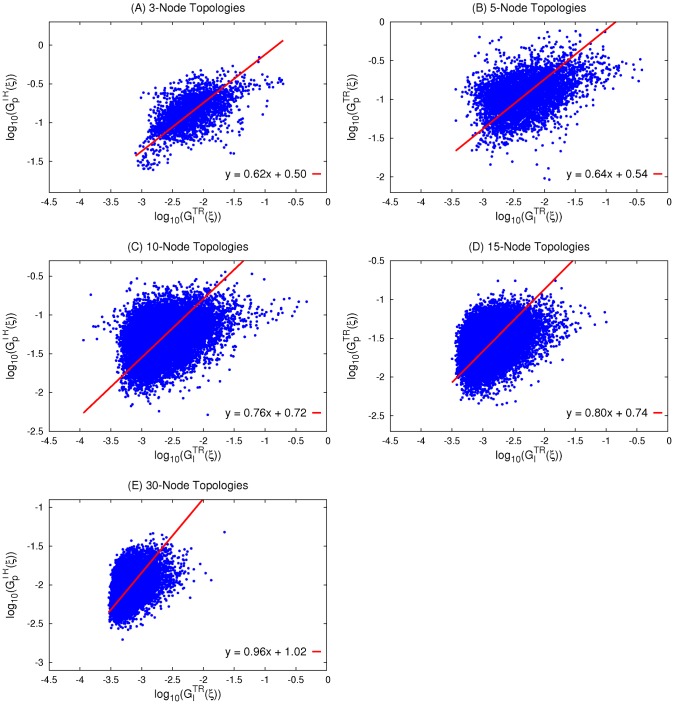
Correlation between robustness to input and parameter perturbations within 3-node, 5-node, 15-node, and 30-node TR networks. 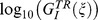
 and 
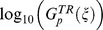
 are measures of robustness of a topology 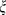 to input and parameter perturbations, respectively, computed from the average over TR networks. Topologies with a low fraction of TR networks (less than 2.3%) are not included. The linear regression for all sizes (3-node, 5-node, 15-node, and 30-node) shows a significant (p<0.0001) correlation between 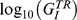 and 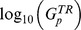. (A) 3-node topologies: slope = 0.62 (*N* = 2445, r = 0.57). (B) 5-node topologies: slope = 0.64 (*N* = 7847, r = 0.40). (C) 10-node topologies: slope = 0.76 (*N* = 18300, r = 0.33). (D) 15-node topologies: slope = 0.80 (*N* = 19264, r = 0.35). (E) 30-node topologies: slope = 0.96 (*N* = 16587, r = 0.41).

**Figure 4:**
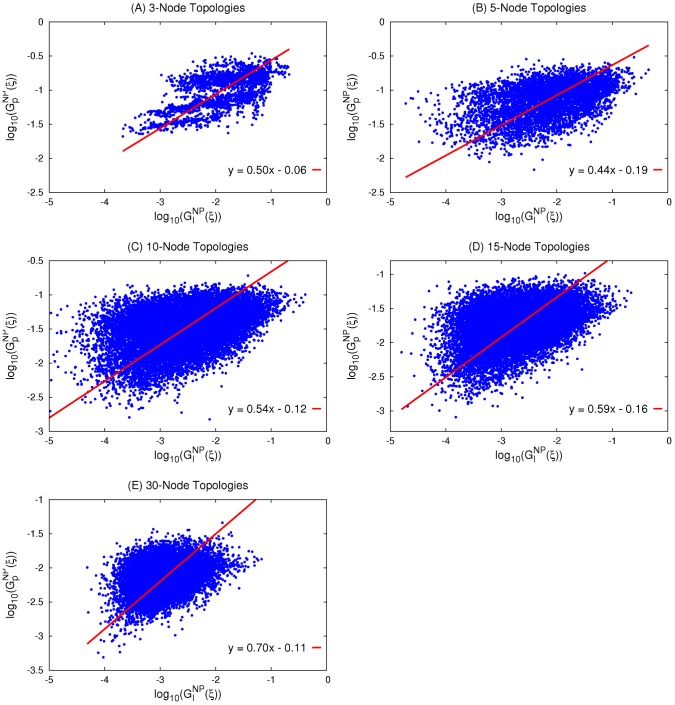
Correlation between robustness to input and parameter perturbations within 3-node, 5-node, 15-node, and 30-node NP networks. 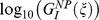
 and 
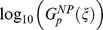
 are measures of robustness of a topology 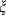 to input and parameter perturbations, respectively, computed from the average over NP networks. The linear regression for all sizes (3-node, 5-node, 15-node, and 30-node) shows a significant (p<0.0001) correlation between 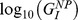 and 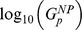. (A) 3-node topologies: slope = 0.50 (*N* = 2445, r = 0.61). (B) 5-node topologies: slope = 0.44 (*N* = 7847, r = 0.47). (C) 10-node topologies: slope = 0.54 (*N* = 18300, r = 0.39). (D) 15-node topologies: slope = 0.59 (*N* = 19264, r = 0.39). (E) 30-node topologies: slope = 0.70 (*N* = 16168, r = 0.30).

For topologies with more than 3 nodes we sample over at least 50000 different ones of each size (5, 10, 15, and 30-node topologies) while 3-node topologies are exhaustively sampled. The different topologies (that have more than 3-nodes) are sampled randomly as described in *Selection Criteria* in the Methods section. We reject topologies with a low fraction of TR networks (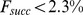, where 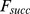 is the ratio of the number of TR networks to the total number of networks) and exclude them from any further analysis. We chose 2.3% to be the cutoff on 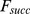 as it is the minimal value of 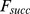 that removes clusters and outliers (Fig. S1). With this, we are left with 2445, 7847, 18300, 19264, and 16589 3-node, 5-node, 10-node, 15-node, and 30-node topologies respectively. The networks sampled from each of these topologies are qualified as TR (Fig. 3) or NP (Fig. 4) and separated accordingly.

We find that over the parameter space of a topology 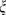, 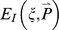 and 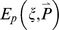 can span a wide range of values. Within both TR and NP networks, we find a significant (p≅0.0) linear correlation between 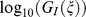 and 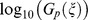. A comparison of the slope of the linear regression (using model II regression, in particular the ordinary least square bisector method described in [52]) shows a clear and systematic pattern between topologies of different sizes and TR and NP networks of the same size. We find that the slope increases as the size of the network increases: slope = 0.62±0.09 for 3-node (Fig. 3A), 0.64±0.05 for 5-node (Fig. 3B), 0.76±0.04 for 10-node (Fig. 3C) 0.80±0.05 for 15-node (Fig. 3D), and 0.96±0.13 for 30-node topologies (Fig. 3E). The marginal error is taken as the 95% confidence interval where the variance of the slope is calculated using its estimate for OLS bisector regression derived by Isobe et al [52]. Similarly, for NP networks we obtain: slope = 0.50±0.04 for 3-node (Fig. 4A), 0.44±0.02 for 5-node (Fig. 4B), 0.54±0.02 for 10-node (Fig. 4C), 0.59±0.02 for 15-node (Fig. 4D), and 0.70±0.03 for 30-node topologies (Fig. 4E). As above, the marginal error here is taken as the 95% confidence interval. The confidence intervals show that, for all sizes, the values of slopes for TR networks are consistently higher than that for NP networks of the same size and that the difference between the two slopes is significant.

The values of the Pearson correlations within TR and NP networks show no clear pattern. This is mainly due to the variability introduced by parameters whose robustness stays invariant and reducible topologies within *N*>3 *N*-node networks (see *Text S1* and *Discussion*). Due to these caveats we are cautious about drawing conclusions based on the values of the Pearson correlation.

### 3-Node Correlations within TR Topologies and Corresponding Motifs

Sampling over all 3^9^ possible topologies, our results show only 4153 topologies have associated TR networks. Within these topologies we find a significant linear correlation before (Fig. S2 A) and after (Fig. 3 A) introducing the cutoff, as discussed in the previous section. In what follows we show how we can extract motifs (i.e., basic topologies that may be more likely to appear in biological systems) by examining the slope of the linear regression between 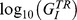 and 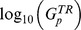. Here, we show that motifs can be extracted from topologies representing the basic backbones shared by a set of topologies showing a stronger relation between environmental and genetic robustness as follows.

**Figure 5:**
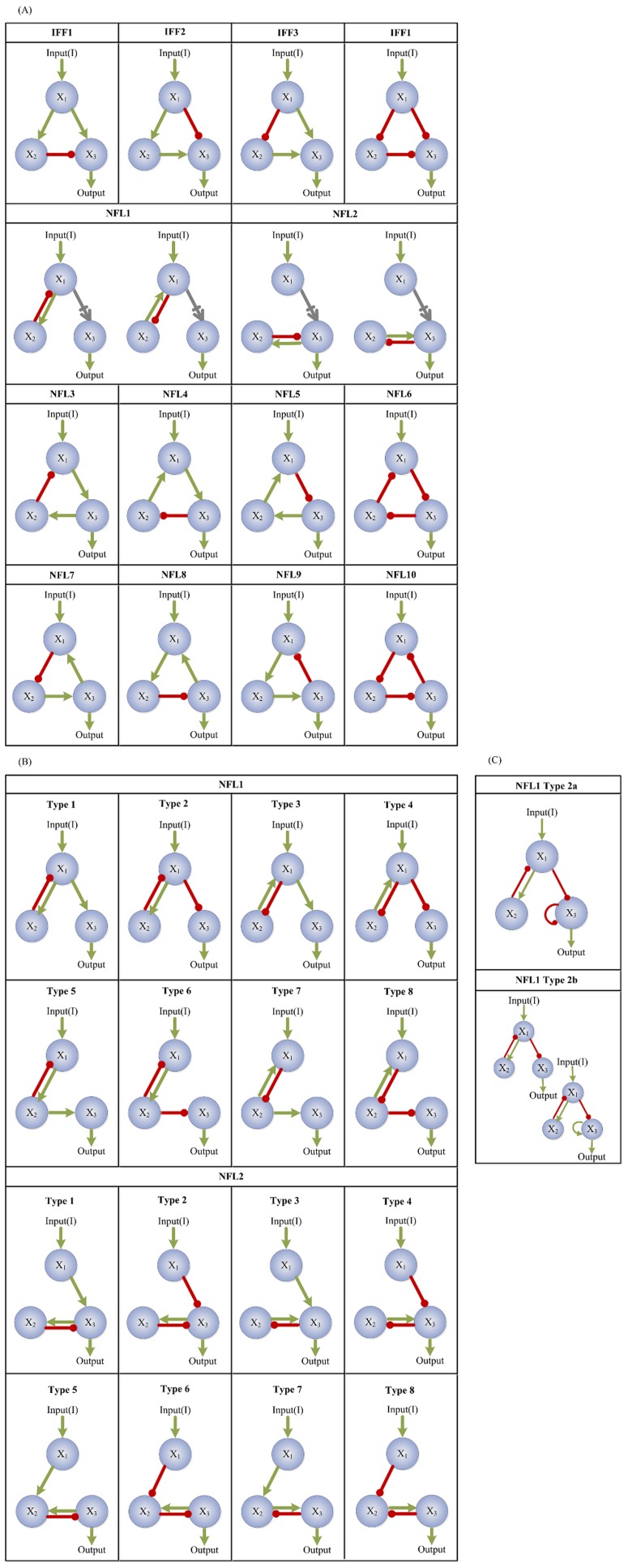
Illustrating the different types of tested motifs. The red, green, and gray arrows indicate deactivation, activation, or either activation or deactivation through a direct or indirect path, respectively. We test two known general motifs, the incoherent feedforward loop (IFF) and the negative feedback loop (NFL). (A) All possible IFF and NFL motifs. (B) All 8 possibilities (types) each for the NFL1 and NFL2 motifs. (C) NFL1 Type2 subtypes.

**Figure 6:**
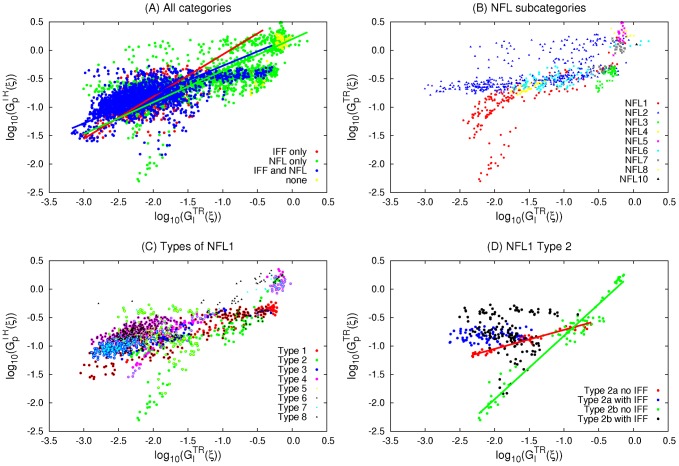
Correlations within 3-node motifs/motif subcategories. Topologies are divided according to the motifs they contain. All the regressions are significant with p<0.0001). (A) Linear regression is applied for each category. IFF_only (red): *y* = 0.74*x*+0.66 (*N* = 609, r = 0.60). NFL_only (green): *y* = 0.56*x*+0.20 (*N* = 1397, r = 0.64). IFF_NFL (blue): *y* = 0.51*x*+0.23 (*N* = 2100, r = 0.63). (B) NFL subcategories excluding topologies that contain more than one motif/type of motif. (C) NFL1 types including those containing additional motifs. (D) Type2a (blue and red)⇒ type 2 with X_3_ auto-deactivation, Type2b (green and black)⇒type 2 without X_3_ auto-deactivation. Type2a without IFF (red): *y* = 0.33*x*–0.38 (*N* = 45, r = 0.97). Type2b without IFF (green): *y* = 1.12*x*+0.29 (*N* = 77, r = 0.97).

We first consider two known motifs, the incoherent feedforward motif (IFF) and the negative feedback loop motif (NFL) and examine their corresponding relations. IFF (Fig. 5A) is a topology wherein the output node is affected by the input receiving node via two paths, one direct and the other indirect, such that, collectively, one path is activating and the other is deactivating. This implies four subcategories denoted IFF1–IFF4. NFL (Fig. 5A) is a topology wherein a node 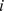 is activated/deactivated by another node 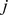, and node 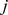 is deactivated/activated back by node 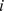 either directly (NFL1, NFL2) or indirectly (NFL3–NFL10). We find that the majority of TR topologies have IFF, NFL, or both IFF and NFL motifs. Only a few have neither IFF nor NFL; these are, however, robust to neither input nor parameter perturbations (Fig. 6A) and they all have low fractions of successful trials, indicating that TR networks generated from these topologies are sparse. All 4 subcategories of IFF are fairly robust to both input and parameter perturbations (results not shown). Though NFL only topologies are generally less robust than those containing IFF, a small group of them (green cluster at the bottom left of Fig. 6A) have low numbers of successful trials but are highly robust within their small TR space. When not coexisting with other robust motifs, only 4 out of the 10 categories of NFL (NFL1, NFL2, NFL4, and NFL6) are robust to both input and parameter perturbations (Fig. 6B). Seeing how NFL1 topologies show separate groups in Fig. 6B, we examine the distribution of all topologies containing NFL1 according to its 8 types (Fig. 5B). We find that NFL1 type1 topologies are strongly correlated (r = 0.92, p≅0) while NFL1 type2 show separate clustering (Fig. 6C). Thus, we further divide NFL1 type2 into two subtypes (Fig. 5C), type2a (the output node deactivates itself) and type2b (all others). While both subtypes show strong correlation between their 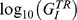 and 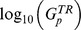 values (Fig. 6D, type2a: r = 0.97 and p = 10^−28^, type2b: r = 0.97 and p = 10^−51^), type2b shows a much steeper slope (1.12 for type2b, 0.33 for type2a, t_test_ = 3.8 and p = 0.0002). This steeper slope may be advantageous for specific biological functions, though both types show strong correlation between the two types of robustness. In the presence of IFF, the two types show no correlation (p = 0.14 and 0.20 for type2a and type2b respectively).

### Fine-Grained Analysis within 3-Node Topologies

In this section, we answer the following questions: (1) What is the reason for the large variation around the regression lines in Figs. 3 and 4? (2) How does the distribution of 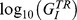 and 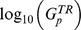 values and their correlation relate to correlations in 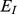 and 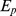 values of the networks within the individual topologies?

To answer the first question, we speculated that since clearly each of the parameters in a topology will have different robustness values, we might be able to separate the parameters into different categories such that the regression along each category leads to different slope values. If we show this to be true, then as the number of possible categories increases, one expects larger variation in the value of 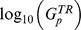 for a given 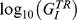 value. If, in addition, the number of categories is proportional to the number of nodes, then the observed variation would increase for a bigger network, as evident in Fig. 3. In what follows, we investigate this possibility within 3-node networks.

**Figure 7:**
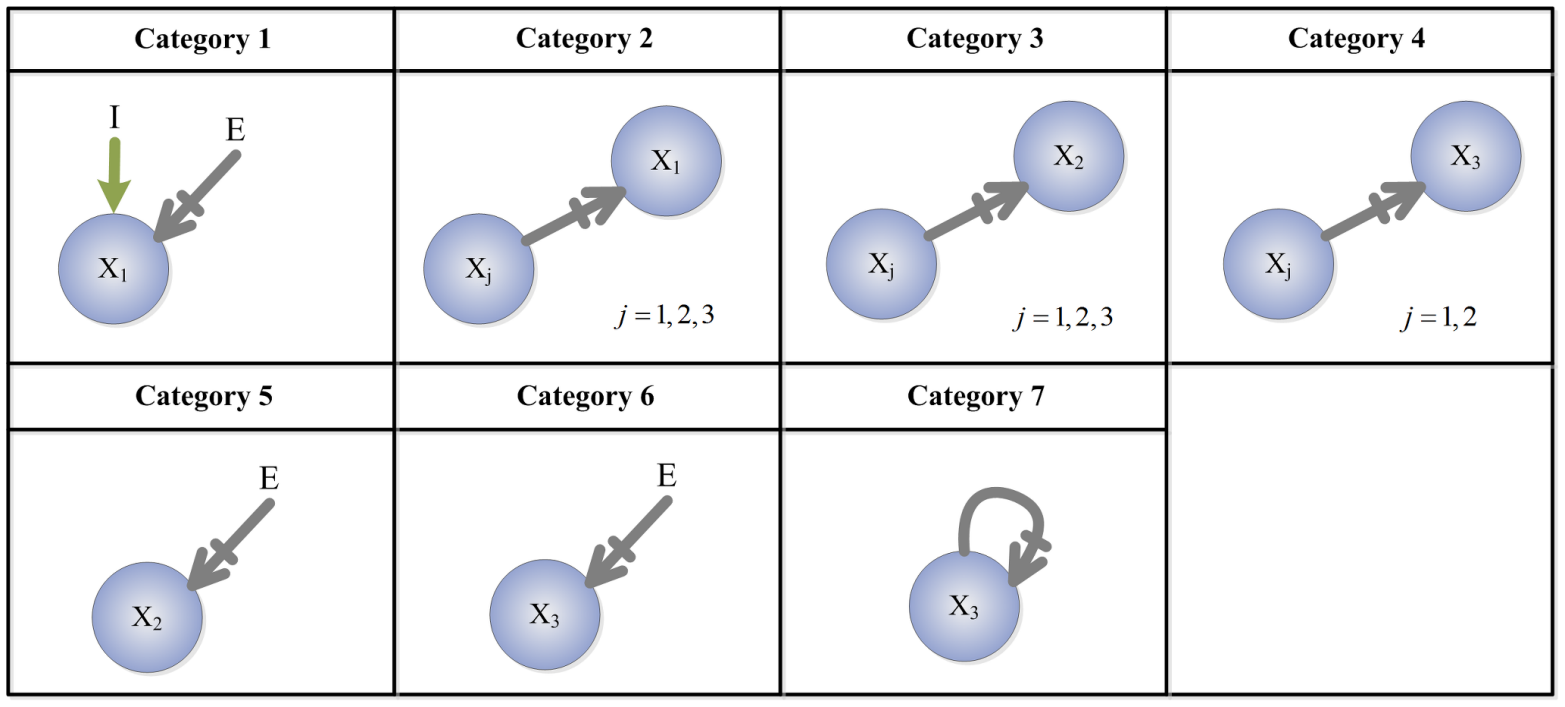
Illustrating the different parameter categories. The links of each topology (and thus their corresponding parameters) are divided into 7 categories: 
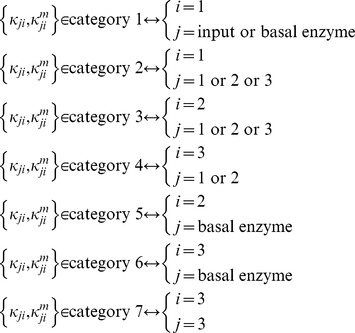

**Figure 8:**
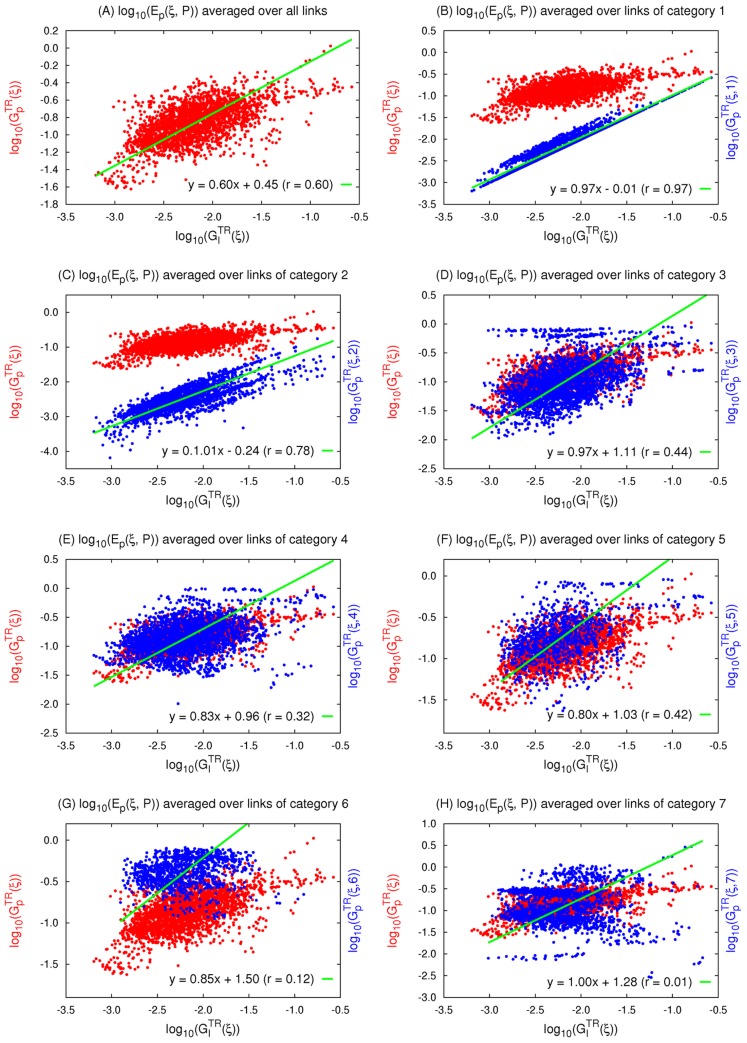
Correlation between robustness to input and parameter perturbations of different categories within 3-node TR networks. 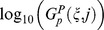
 is a measure of robustness of topology 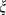 to perturbation in parameters 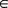 category 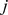. Topologies with a low fraction of TR networks (less than 2.3%) are not excluded. We show the linear regression between robustness to perturbations in input and in (A) all parameters, (B) parameters 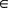 category 1, (C) 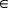 category 2, (D) 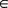 category 3, (E) 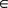 category 4, (F) 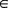 category 5, (G) 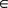 category 6, (H) 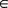 category 7. The linear regression results are shown on the Figure. All linear correlations are significant (p<0.0001) except for category 7 (H) slope = 1.0 but r = 0.001 and p = 0.81.

Consistent with the 5-, 10- 15-, 30-node analysis above, we remove topologies with a low fraction of TR networks (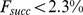) and are left with 2534 topologies to work with.. Next, we separate the parameters of each topology into 7 categories (Fig. 7). Parameters belonging to categories 1 or 2 are those associated with links affecting (i.e., directed towards) the input receiving node, node 1. Those belonging to categories 3 or 5 are associated with links affecting the buffer node, node 2. The rest (in categories 4, 6, 7) are associated with links affecting the output node, node 3. Then, for each category *j* of a network 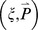, we evaluate the value 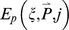 which takes into consideration only robustness to perturbations in parameters belonging to category *j* (Eq. 26 in *Methods*). The corresponding value for the topology 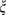, is 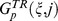 (Eq. 28 in *Methods*). We find that indeed the regression on each of the 7 categories results in a different slope and different correlation strengths. The results of the overall linear regression are shown in Fig. 8A. For the separate categories, we find that the strongest correlation is between 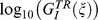 and robustness to perturbations in parameters belonging to category 1, 
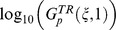
 (Fig. 8B, slope = 0.97, r = 0.97, p = 0), followed by category 2 (Fig. 8C, slope = 1.01, r = 0.78, p = 0). Conversely, 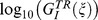 and 
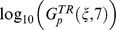
 show no correlation (Fig. 8H, slope = 1.0, r = 0.01, p = 0.81). In fact, the strength of the correlation between 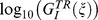 and 
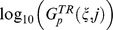
 decreases in the following order: j = 1 (r = 0.97), 2 (r = 0.78), 3 (r = 0.44), 5 (r = 0.42), 4 (r = 0.32), 6 (r = 0.12), and 7 (r = 0.01).

The second question is related to whether within each topology the parameter subspace corresponding to input robustness is positively correlated with that corresponding to parameter robustness. If they are not correlated, then the two subspaces could be disjoint and the collective/coarse-grained correlation (i.e., the correlation between the 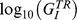 and 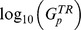) does not support our hypothesis.

**Figure 9:**
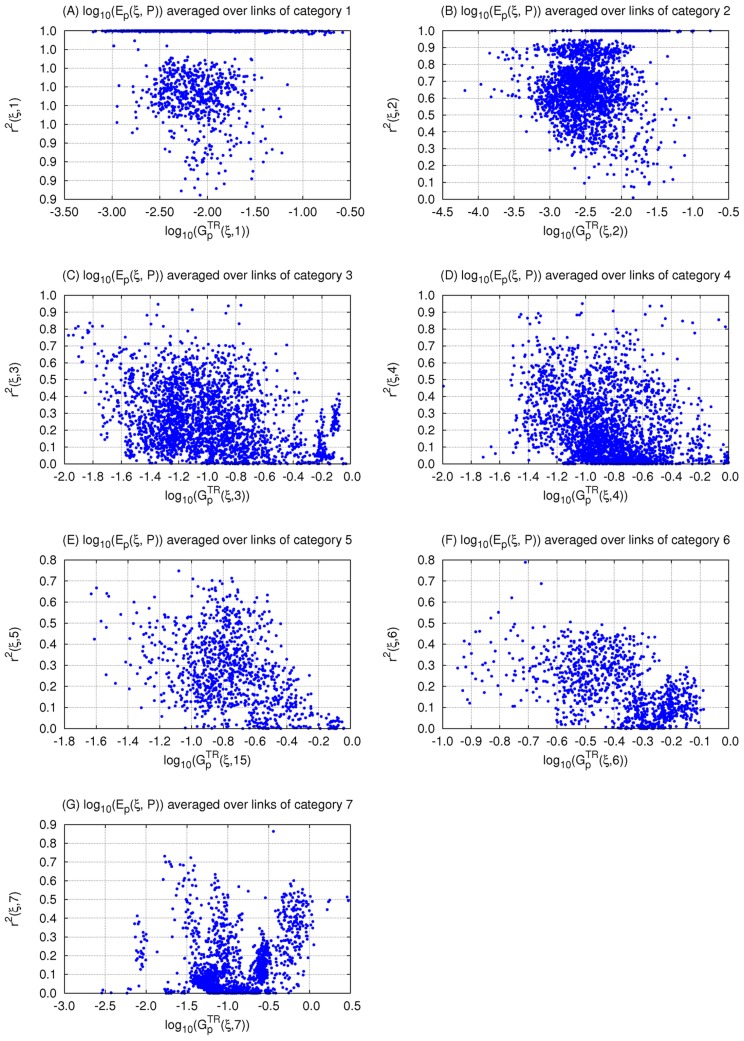
r^2^ within the networks of each 3-node topology divided into 7 categories. Within each topology 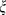, the overall robustness to parameters in category 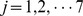 is shown versus 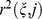, the *r^2^* of the correlation (i.e., square of the Pearson correlation) between 
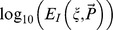
 and 
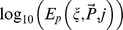
, for *j* = 1 (A), 2 (B), 3 (C), 4 (D), 5 (E), 6 (F), and 7 (G).

**Figure 10:**
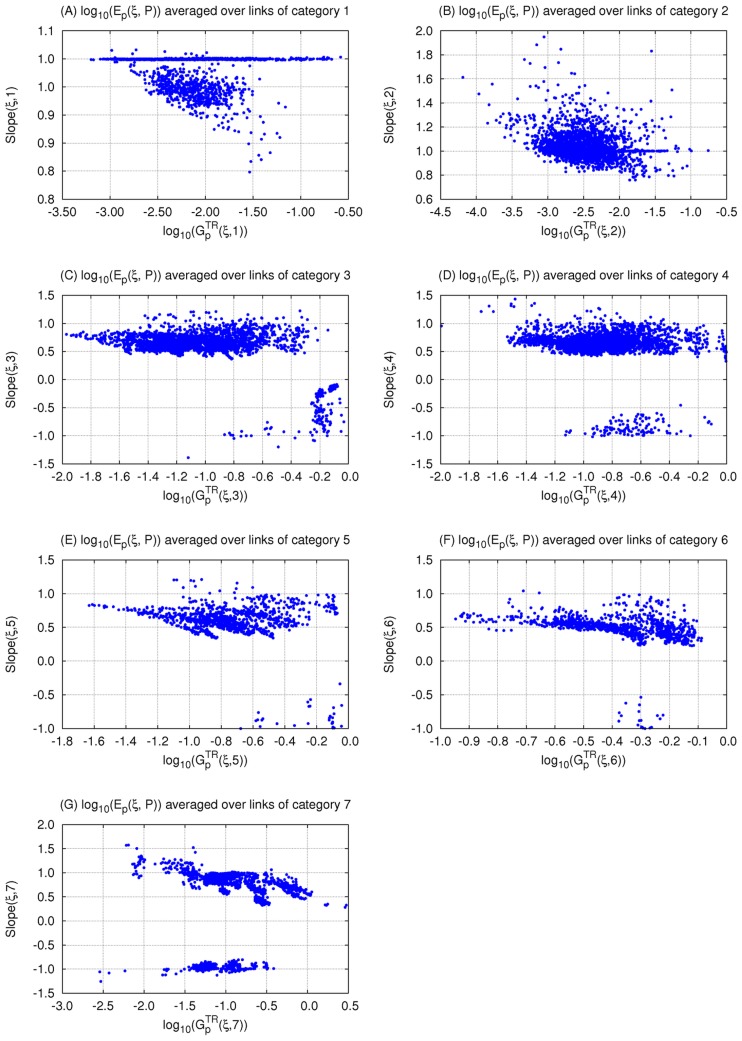
The slopes within the networks of each 3-node topology divided into 7 categories. Within each topology 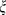, the overall robustness to parameters in category 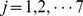 is shown versus 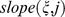, the slope of the regression line between 
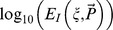
 and 
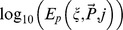
, for *j* = 1 (A), 2 (B), 3 (C), 4 (D), 5 (E), 6 (F), and 7 (G).

We follow the same procedure as above and separate the parameters into the 7 categories depicted in Fig. 7. The aim is to be able to compare the results with those in Fig. 8. For each topology 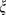, we perform a linear regression on the relationship between 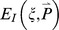 and 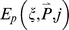 for each category *j*. The results of the correlation strength and slopes are represented by their corresponding square of the Pearson correlations 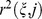 and slopes 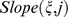, for 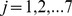. The relationship between 
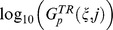
 and 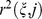 is shown in Fig. 9 while that between 
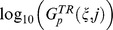
 and 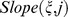 is shown in Fig. 10. As above, the strongest correlations and steepest slopes are found between 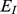 and 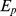 of parameters belonging to category 1, 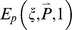. For all the topologies, 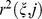 remains ≥0.9 (Fig. 9A) and 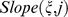 ≥0.8 (Fig. 10A). A weaker fine-grained correlation indicates a less collective robustness as indicated by the increase in 
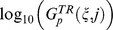
 (i.e., decrease in parameter robustness) as 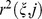 decreases, for 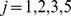 (Fig. 9A,B,C,E). This pattern does not appear for 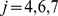 (Fig. 9D,F,G), which is consistent with the results in Fig. 8 where categories 4, 6, and 7 show the weakest correlations between 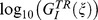 and 
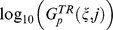
 compared to the other categories. In particular, most of the 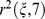 values are very small, less than 0.2, which is consistent with the results in Fig. 8H where no correlation is found (as indicated by the high p value). Furthermore, one can map the different clusters appearing in Fig. 8B–H into the clusters that appear in Fig. 10A–G. For example, the set of topologies showing a 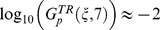 can be mapped to the cluster in Fig. 9G with 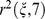 ranging between 0 and 0.4 and that in Fig. 10G with 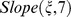 ranging between 1.0 and 1.5. Similarly, in Fig. 8D, the separate two sets of topologies showing a low parameter robustness value (
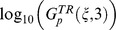
 between −0.2 and 0) can be mapped to the two clusters in Fig. 9C on the top left side with 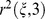 ranging between 0 and 0.3 for one, and between 0.2 and 0.4 for the other, and the two clusters in Fig. 10C with negative values of 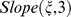. Further investigation of the set of topologies corresponding to the different clusters goes beyond the scope of the work presented here.

### Plausible Models of the Role of CheV-P in Bacterial Chemotaxis

**Figure 11:**
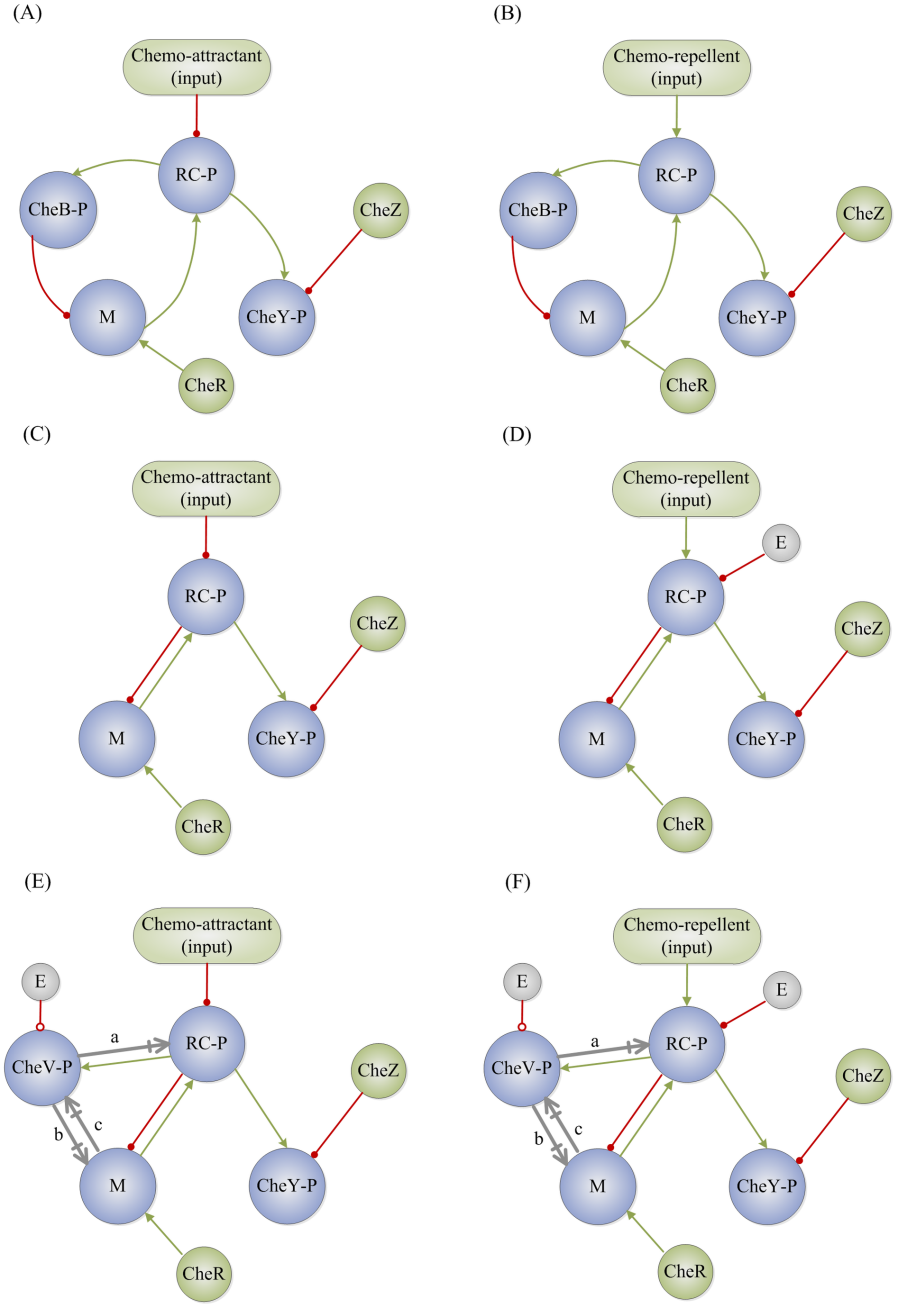
Chemotaxis biochemical adaption networks. (A) and (B) are the original networks of E. coli chemotactic adaptation as described in the literature [17]–[21] under positive (A) and negative (B) stimuli. (C) and (D) are their respective coarse-grained topologies. (E) and (F) are the coarse-grained networks involving phosphorylated CheV. The grey edges (a, b, c) are putative additional interactions. They can be activating (+1), deactivating (−1), or have no effect (0) as listed in table 1.

The main proteins/receptors involved in E. coli chemotaxis are CheA, CheW, CheB, CheR, CheZ, and CheY. E. coli uses an anticlockwise rotation of its flagella to move forward. A decrease or increase in the concentration of nutrients (chemo-attractants) or harmful chemicals (chemo-repellents), respectively, provokes a change to a clockwise rotation which causes the E. coli to tumble and thus change direction. This signal to the flagella is controlled by the chemotaxis protein CheY. A stimulus (i.e., a change in the chemical concentration in the environment) is sensed by periplasmic binding proteins which couple to CheA in the inner membrane with the help of CheW. An increase in chemo-attractant concentrations inhibits the phosphorylation of the receptor complex CheA-CheW (RC-P) (Fig. 11A) while a chemo-repellent enhances it (Fig. 11B). RC-P gives its phosphate group to both CheY and CheB (CheY-P, CheB-P). CheB-P demethylates glutamate residues while CheR enhances methylation. In turn, methylated glutamate (M) enhances the phosphorylation of the receptor complex. The chemotaxis protein CheZ helps speeding the autodephosphorylation of CheY-P [19]–[21] (Fig. 11A–B). For simplicity, we further coarse-grain this network such that M and RC-P interact via a negative feedback loop (Fig. 11C–D). In the supplementary material (Fig. S3), we demonstrate that there is no significant difference in the results between the topologies shown in Fig. 11A and its coarse-grained equivalent shown in Fig. 11C (slope = 0.79 and 0.77, respectively), though coarse-graining improves the Pearson correlation as it removes the redundant link leading to additional variability. The topology under the influence of a chemo-repellent has a much lower fraction of TR networks and shows no correlation (r = −0.01, p = 0.85) in its un-coarse-grained form (Fig. 11B). It was important to remove the redundancy to obtain a significant correlation (Fig. 11D, slope = 0.37, r = 0.45, p = 10^−14^).

Chemotaxis in many other bacteria is more complex and involves more proteins. One such protein is CheV which generally contains a phosphorylatable domain [24]. Here we consider all possible coarse-grained interactions between phosphorylated CheV (CheV-P), RC-P, and M. The only assumption we make is that RC-P gives its phosphate group to CheV in addition to CheB and CheY (Fig. 11E–F). With this, we obtain 3^3^ possible sets of signed directed edges as listed in table 1, where we are considering all 3 possibilities (i.e., activation, deactivation, or no link) for the 3 suggested links.

**Table 1:** All possible sets of interactions for links a, b, c in Fig. 11.

Index	a	b	c
**1**	1	1	1
**2**	1	1	0
**3**	1	1	−1
**4**	1	0	1
**5**	1	0	0
**6**	1	0	−1
**7**	1	−1	1
**8**	1	−1	0
**9**	1	−1	−1
**10**	0	1	1
**11**	0	1	0
**12**	0	1	−1
**13**	0	0	1
**14**	0	0	0
**15**	0	0	−1
**16**	0	−1	1
**17**	0	−1	0
**18**	0	−1	−1
**19**	−1	1	1
**20**	−1	1	0
**21**	−1	1	−1
**22**	−1	0	1
**23**	−1	0	0
**24**	−1	0	−1
**25**	−1	−1	1
**26**	−1	−1	0
**27**	−1	−1	−1

**Figure 12:**
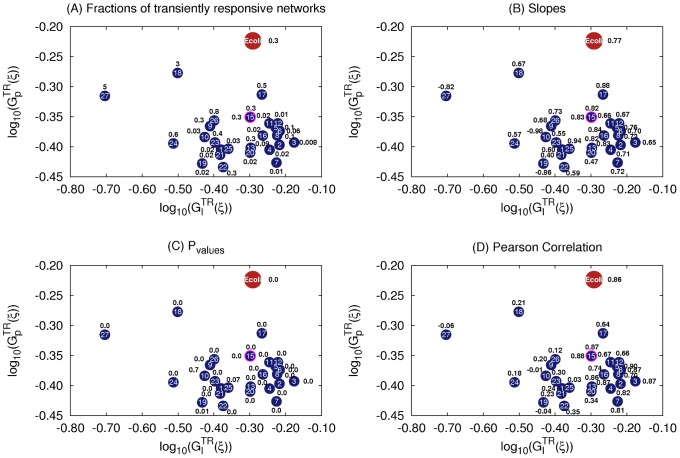
Chemotaxis networks responding to a change in a chemo-attractant. Each blue circle represents a topology responding to change in the concentration chemo-attractants in the environment. The index of each topology is given inside the circle. The blue circle highlighted in red represent two topologies, 14 and 15, whose positions overlap. The corresponding values of the fraction of TR networks (A), slopes of the linear regression of their 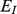, 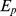 values (B), p (C), and r (D) values of the regression are shown next to the blue circle.

**Figure 13:**
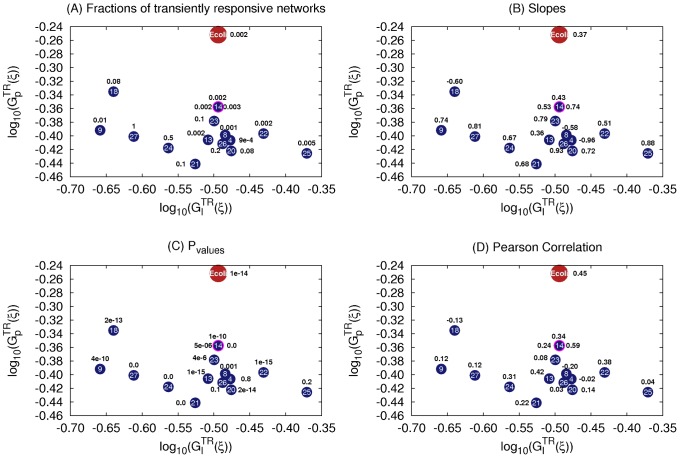
Chemotaxis networks responding to a change in a chemo-repellent. Each blue circle represents a topology responding to change in the concentration of chemo-repellents in the environment. The index of each topology is given inside the circle. The blue circle highlighted in red represent three topologies, 14, 15, and 17, whose positions overlap. Their corresponding values of fraction of TR network (A), slopes of the linear regression of their 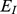, 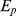 values (B), p (C) and r (D) values of the regression are shown next to the blue circle.

For each of the 27 topologies, we compute the 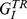 and 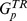 values (Figs. 12, 13) and the slopes of the regression between 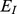 and 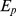 values of their corresponding TR networks (Figs. 12B, 13B). We compare the results with that of the E. coli topology both under positive (Fig. 12) and negative (Fig. 13) stimuli. Topologies 1–3, 5–7, 10–12, 16, and 19 are highly improbable as they have no significant number of TR networks within the sampled parameter space when chemo-repellents are the stimulus (Fig. 13). Topologies 4, 8, 18, 25–27 are also eliminated as they either show either a negative or no correlation (Fig. 12C–D, 13C–D) under either a chemo-attractant or a chemo-repellent. Topologies 9, 14, 18, 21, 23 are less likely than the rest (13, 15, 20, 22, 24) as they have a weaker correlation between 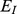 and 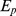 than E. coli as deduced from the lower p values (Fig. 12C, 13C). Topologies 20 and 22 are less robust to input perturbation than Ecoli when chemo-repellents are the stimulus (Fig. 13), and 24 has a significantly smaller slope. Finally 13 is more robust to parameter perturbations than 15. The distributions of 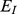, 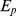 for each topology are shown in Figs. S4, S5, S6.

## Discussion

In this work, we demonstrated that there is a general positive power-law correlation between environmental and genetic robustness in TR networks, and a statistically significant trend to a directly proportional linear relationship between the two in the limit of large networks. Conversely, monotonically responsive and non-responsive (NP) networks show a weaker relationship than TR ones. Furthermore, this distinction between the two classes becomes more prominent as the size of the networks increases. Therefore, this relationship associated with TR may be relevant to the evolution of biochemical networks. While other factors have played a role in the evolution of genetic robustness, our results show that, for TR networks, as the system evolves to withstand external environmental perturbations, it will, with high probability, concomitantly become robust to certain genetic perturbations.

We speculated that the inverse of the slope is proportional to 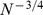 where 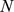 is the number of nodes. We performed the corresponding regression and obtained 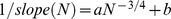 for 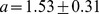 and 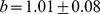 (r = 0.9439_,_ p = 0.008 (1-tailed), p = 0.016 (2-tailed)). To confirm our results, we performed a Bayesian analysis for the model 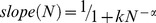 with a uninformative flat prior on the parameters and obtained 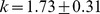 and 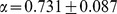 from the second moments of the posterior. Thus the Bayesian analysis confirms the linear regression. For NP networks, the same regression gives 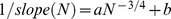 for 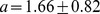 and 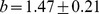 (r = 0.7608_,_ p = 0.07 (1-tailed), p = 0.13 (2-tailed)). The value of 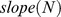 for TR networks in the limit of *N* large is thus 0.99±0.08 while that of NP networks is 0.68±0.21. While the latter's regression is not significant at the p = 0.05 level, the value of the intercept did not significantly change for different power values (we tried 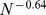 and 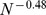). The statistically significant regression for TR networks implies that as a network evolves to be more robust to input perturbations it will also evolve to be robust to parameter perturbation (and vice versa) at a faster rate. Most importantly, as the size of TR networks becomes larger, the linear relationship between the logarithms quantifying robustness to input and that to parameter perturbations implies that for larger TR networks, 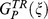
is, with statistical significance, and within the computed uncertainty, proportional to 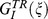 while for larger NP networks, 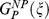 tends to be proportional to 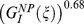. As standard in the analysis of power-law relationships, we computed the regression using logarithms. For specific biological situations, it may be conceptually more appropriate to compute a direct fit, but for general random networks, we know of no such principle. An exponential fit between 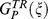 and 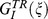 for different numbers of nodes would be difficult to interpret as the power-law is changing with the number of nodes, tending to a constant only as the number of nodes becomes large.

A drawback of our method is that the random generation of large networks does not account for reducible topologies which can introduce more variability and thus more error and a lower correlation between the two robustness measures. This makes a comparison between the correlation coefficients of topologies of different sizes a trifle problematic. However, the space of topologies grows so rapidly with the number of nodes that the likelihood of randomly selecting a reducible network decreases precipitously. Similarly, the averaging method does not distinguish between links contributing to the robustness of either input or parameters and those that do not. A method that could pinpoint such links would be useful in this context.

Our results on the adaptability of 3-node motifs differ somewhat from Ref [30] due to our use of a qualitative test, the Pearson shape correlation, for assessing the transient response property of a network. We are not aware of a biologically plausible rationale for an explicit cutoff on the size or speed of a response as biological examples can exhibit both extremes of size or duration of transients. The general motifs shown in the literature [30] need further qualification to be deemed biochemically adaptable. For example, many topologies containing NFL are nonresponsive. Conversely, we show that a subcategory of NFL, NFL1 type2b is particularly robust and exhibits a strong correlation between robustness to input and parameter perturbations (Fig. 6D).

Our results are consistent with biological networks described in the literature. For example, we show that the coarse-grained network topology of E. coli chemotaxis, as described in the literature [17]–[21], is NFL1 type2b (Fig. S7C), as follows. When the receptor complex is activated, it causes the phosphorylation of the response regulator CheY leading to increased probability of tumbling. An increase in the chemo-attractant level (*I*) suppresses the activity of the complex and, in turn, the phosphorylation of CheY (Fig. S7A). If *I* is the input (which we set to always activate the input-receiving node in our computations, for consistency), then we can define the concentration of the input-receiving node as that of the deactivated complex, *X_1_* (i.e., the activated complex represent *X_1_* in its deactivated form). In this case, *X_1_* deactivates CheB which inhibits methylation (*M*). *M* activates the complex which is equivalent to deactivating *X_1_*. The latter inhibits the phosphorylation of CheY (the output) and thus decreases the probability of tumbling (Fig. S7B).

An example of IFF is the Ras model of MAPK cascades discussed in Ref [53]. The input simultaneously activates two factors, SOS and RasGAP which activate and deactivate Ras, respectively and simultaneously. The model is shown [53] to be responsive only when the activation of SOS is faster than that of RasGAP. Thus, one can further coarse-grain it by removing the intermediate node between Ras and the input node (Fig. S8). This reduces to an IFF1 topology.

In Ref [30], all NFL topologies wherein the output node directly affects the input receiving node were found to be not robust or transiently responsive. While consistent with our results showing that NFL7–NFL10 are not robust, note that when the negative feedback loop has a direct and an indirect path, the outgoing and incoming links of the input receiving node must have the same sign for adaptability and parameter robustness to be achieved (see NFL4 and NFL6 as opposed to NFL3 and NFL5 in Fig. 6B). Our work goes beyond pointing out general motifs. We refine subcategories within these motifs and show that, in fact, they do vary in their biochemical adaptation properties.

Traditionally, network motifs represent subgraph topologies that appear in biological networks much more often than one would expect in a randomly constructed network [49], and specific functions were assigned to different types of motifs [41], [46]–[48]. The validity of this approach has been questioned as the frequency of occurrence of these motifs was not statistically significant when compared with corresponding (i.e. same degree) randomly constructed networks [54]. It was argued that one cannot analyze subgraphs independently of the rest of the network as interactions will drastically change the functions assigned to the particular topology [55]. In our work, a motif does not represent a subgraph, rather the topology of the backbone of (possibly much) bigger networks.

We use our approach to differentiate between plausible models of the role of the CheV-P protein in bacterial chemotaxis. We find that there are only a few possible ways that CheV-P can be linked to RC-P and M. We suggest that while there are at most 9 possible topologies, the most plausible one has M enhancing the phosphorylation of both CheV and the receptor complex.

Some specific network features have been associated with robustness to environmental variation in bacterial gene expression. Insulating gene expression by different modes of control, from activation to repression depending on the required high or low activity, has been suggested as a general control feature [56].

Our approach to motif discovery can be extended to networks with backbones with more than 3 nodes. While exhaustive enumeration of small motifs with desired functions is fascinating [30], [41]–[43], it is neither immediately evident nor has it been demonstrated in any context that such motifs could be put together to make systems with multiple functions while preserving the robustness or responsiveness properties of the separate motifs. To get to the point where we can plausibly discuss architectural principles in biology, it seems necessary to find general characteristics of classes of networks of all sizes that could perform functions of biological interest. Our work is a step towards this goal.

## Methods

### Notations

Following the same initial setup as in Ref [30], a biochemical network is represented with a directed signed graph 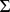 wherein the nodes of the network represent the enzymes. The latter can either be active or inactive and are able to interconvert between the two states. Thus, the elements of the corresponding adjacency matrix 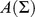 can take the values 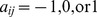, implying that node 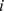 deactivates node 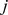, has no effect on 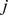, or activates 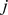, respectively. No parallel links going in the same direction are allowed, i.e., 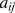 cannot be >1. We divide the nodes into two types, varying nodes and fixed nodes. The latter correspond to inputs and basal enzymes which are added to each network to ensure that each node has at least one activating and one deactivating link. Thus, for an *N*-node network with 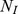 inputs and 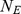 basal enzymes, 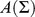 is an 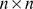 matrix where 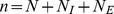. These concentration values are represented by an 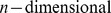 vector
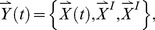
(1)where 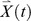 is the concentration of the active form of the 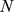 enzymes at time 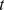,
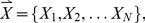
(2)

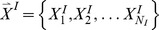
 and 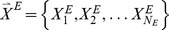 are the time-independent concentrations of the 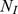 inputs and 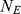 basal enzymes, respectively.

Assuming that the enzymes are non-cooperative and hence that they obey the Michaelis-Menten kinetics, the rate equations governing the dynamics of the network take the following compact form
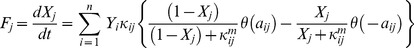
(3)where 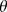 is a unit step function defined as
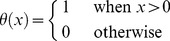
(4)

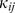
 and 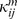 are the catalytic and Michaelis-Menten rate constants for the regulation of enzyme 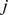 by enzyme 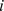, for 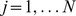 and 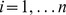.

In equation (3), the total concentration of each enzyme is kept constant and normalized (i.e., the concentration of the active form of an enzyme plus that of its inactive form is always equal to one). Thus, 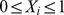 for 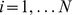. For all simulations presented here we use only one input, 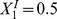. This particular choice of input concentration should not have a significant effect on our qualitative results, as we have checked explicitly while formulating our hypothesis. Networks are allowed to reach steady state before the concentration of the input is perturbed. We are only concerned with the relative change in steady state concentrations.

### Experimental Setup

*N*-node networks are identified with directed signed graphs 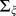 representing their topology and a set of parameters 
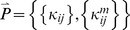
, for 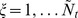, where 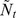 is the total number of sampled topologies, excluding those wherein one or more nodes have a total degree of zero or the output node cannot be reached from the input receiving node (Fig. 1). Each topology, in turn, is sampled over a large number of random networks, i.e., a large number of randomly chosen parameter sets 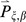, for 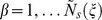, where 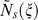 is the total number of sampled networks (sets of parameters) for topology 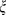. The total number of parameters in each set 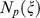 (i.e., length of the vector 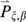) varies depending on the topology. The order of magnitude of 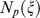 increases exponentially with the size of the networks. For example, 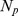 values for 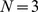 are around 20, around 40 for 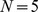, and 500 for 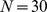. Similarly, the number of iterations (i.e., number of sampled networks 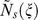) required (see *Selection Criterion* below) also increases with 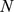. For example, for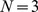 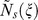 values range between 10^4^ and 10^5^, while for 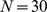, 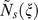 values range between 10^6^ and 10^7^. Typically, an iteration takes less than 10^−3^ seconds of CPU time for small networks (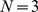), thus 1 to 2 minutes to test each topology. On the other hand, for large networks (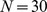), an iteration typically takes around 0.04 seconds of CPU time, 3 to 5 days for testing each topology.

### Pearson Test

We define a TR network as one whose output dynamics has a non-monotonic transient between two steady states as a response to input change (i.e., the steady state values before input perturbation and that after input perturbation). We find the transition time (i.e., the time at which the concentration is maximal/minimal before it starts decreasing/increasing again) and enzyme concentrations, 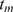 and 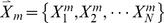 (i.e., concentrations at 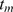), by solving for the turning point
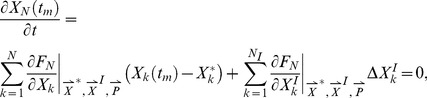
(5)where 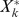 is the concentration of node *k* at steady state.

We use a Pearson test to determine if a given network is TR.

First, we define two functions 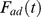 and 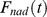 as model functions of perfect adaptability and non-adaptability (a monotonically changing network), respectively (Fig. 2):
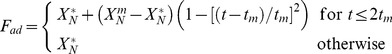
(6)

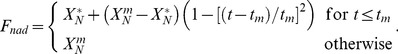
(7)Define corresponding Pearson shape correlations 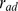 and 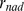 as
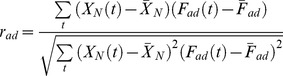
(8)

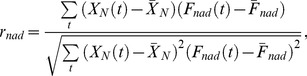
(9)where 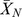, 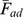 and 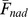 are the mean values of 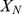, 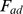 and 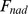. With this, a network is deemed TR if 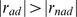. Comparing the absolute values or 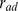 and 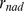 instead of the actual values is necessary. Even though our definitions of 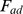 and 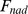 will most likely lead to positive values, this is not always the case. The reason is that eq. (6) and (7) assume the perfect case where the differences in the concentrations from the initial steady state have always the same sign (as in Fig. 2 E and F). If instead the difference in concentrations at the transition point is smaller than that at the final steady state (i.e., post-perturbation steady state), then 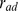 and/or 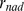 will have negative values. However, that does not matter since we are only interested in the shape of the time-course (see Fig. 2G, for example).

**Figure 14:**
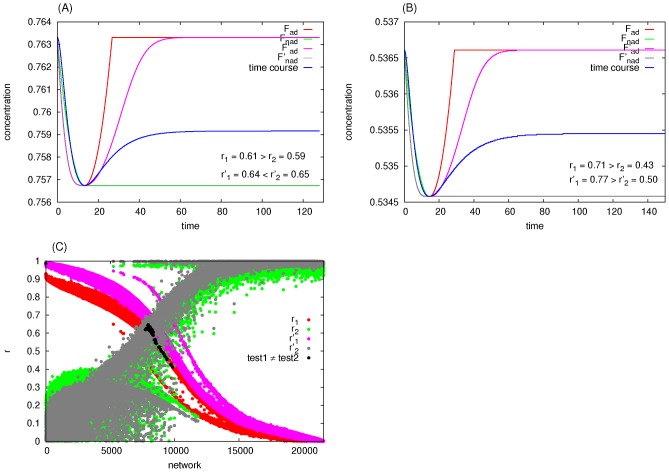
Variations in the Pearson test within individual networks. When the Pearson test is performed using two different definitions of 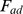 and 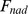, small variations can be observed within individual networks. In (A) we show an example where a network's time-course (blue) is deemed TR when the definition, 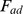 (red) and 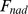 (green) of equations (6) and (7) is used (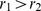), but NP when the definition, 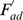 (pink) and 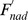 (gray) of equations (10) and (11) is used instead (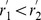). (B) is an example where the two tests agree (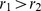 and 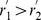). In (C) we show all 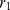 (red), 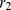 (green), 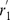 (pink), and 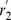 (gray) values over the space of networks. Networks where the two tests (i.e., Pearson test using the two different definitions) differ are shown in black (note that only their 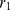 values are colored in black to avoid showing the same network 4 times).

Note that the mean values are taken as the average over all the discretized time-steps; for example, 
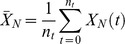
 for 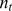 time steps. The size of the time-step, 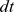, is the same for all networks (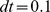), but this is not the case for the number of time steps, 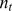, as the length of the time-course of each network varies depending on how long the network needs to reach a new steady-state (i.e., the rate equations in eq. (3) for all nodes reach zero again after input perturbation. Of course, computationally, the run will stop when the rate equation for all nodes is less than 10^−10^). For example, in Fig. 14A and 14B we show the time-courses (in blue) of two different networks. The network in Fig. 14A needed around 130 seconds (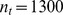) to reach a steady state, while that shown in Fig. 14B needed around 150 seconds (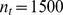). Simulations that take too long to reach a steady state (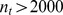) are thrown away and not considered in the analysis (i.e., are thrown away without performing the Pearson test). This cutoff on the maximal number of time-steps allowed is chosen for computational efficiency. Preliminary results showed that for most networks, if a steady state was not reached within 2000 time-steps, it is unlikely it will be reached for a long time. Since we are only interested in the statistical results and since networks are chosen randomly, there is no reason to insist on including a network that takes a lot computational time to reach a steady state. We chose 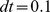 because we were looking for the largest time-step (to improve computational time) that does not change the statistical results. In preliminary runs, we compared the results of 3-node networks when using 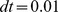 and 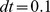. The finer time-step allowed more topologies to pass as TR. However, these topologies had very low fraction of TR networks and were removed after the cutoff. Moreover, the statistical results were the same both before and after the cutoff. As mentioned in *Experimental Setup* above, large networks (30-node) take 3 to 5 days of CPU time for each topology. Using 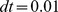 would increase this simulation time to over a month for each topology which is impractical.

### Robustness of the Pearson Test

We test the robustness of the Pearson test described above by comparing the results from the 3-node simulations to those employing instead the Spearman correlation using the same definition of 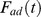 and 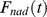 (Fig. S9A). Both are also compared to simulations using a different definition,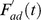 and 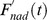 as follows:
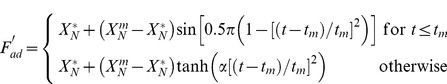
(10)

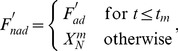
(11)where 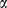 is chosen here to be 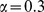. This new definition allows 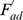 and 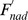 to get to the transition concentration, 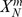, at a slower rate, then after the transition point, 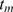, 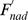 coincides with 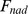 while 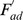 relaxes back to the pre-perturbation steady state, 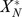 at a much slower rate (Fig. 14A,B). In all cases we find no significant difference between the results for 3-node simulations (Fig. S9). This does not mean that there are no variations within individual networks. For example, in Fig. 14, we show the 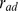 and 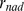 values of the Pearson test for all the networks corresponding to a typical 3-node topology using both definitions (Fig. 14C). We find that there are 90 out of 21579 networks that were deemed TR in one but NP in the other. A typical time-course where the outcome of the Pearson tests differ or agree are shown in Fig. 14A and Fig. 14B, respectively. In general, most networks do not fall into this category where the values of 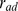 and 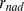 are very close such that different definitions of 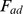 and 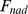 lead to different outcomes. In Fig. S9, we verify that this change does not affect any statistical observations.

### Quantitative Measure of Network Robustness

To quantify the degree of robustness to input and parameter perturbations of a particular network, we calculate the relative change in the steady state concentrations of the output node due to perturbing the input and parameter values, respectively. Let 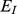 and 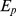 be the average of the sensitivity of the steady state concentration of the output to each input and each parameter, respectively. Then,
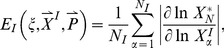
(12)

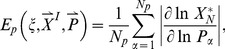
(13)where the 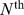 node is the output node, 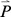 is the set of 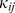 and 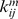 corresponding to each reaction/link (i.e, the non-zero values), 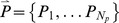, 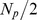 is the total number of links, and 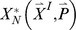 is the set of steady state concentrations of the output node for input 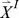 and parameter set 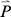.

Defining the degree of input and parameter robustness of a network 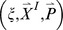 as inversely proportional to the values of 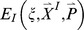 and 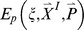 ensures all the inputs and parameters of the network are taken into consideration. Analyzing the rate functions of equation (3) (see *Steady State Analysis* in the *Text S1* for the detailed derivation), we obtain
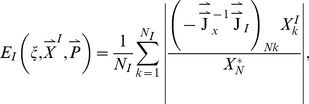
(14)

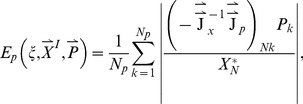
(15)where 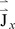, 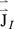, and 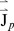 are the Jacobians with respect to the node concentrations, input, and parameters, respectively.

### Quantitative Measures of Robustness of TR Topologies

A robust topology is one that gives rise to robust networks with a higher probability when tested with a large number of parameter sets. Quantitatively, the degree of robustness to input perturbations of a given topology is taken to be the geometric average of 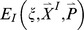 over all 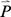. Similarly, the degree of parameter robustness is the geometric average of 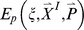. A TR topology is one that has a statistically significant number of TR networks. Topologies that do not have enough TR networks are rejected and excluded from any further analysis. With this, we are left with 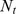 topologies out of the 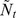 sampled ones. For each topology 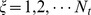, we define two quantitative measures each for input (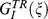 and 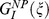) and parameter (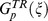 and 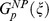) robustness. 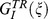 and 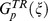 are the values of input and parameter robustness of TR networks
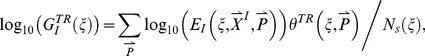
(16)

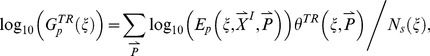
(17)

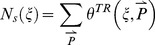
(18)where 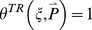 if network 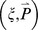 passes the Pearson test and zero otherwise.


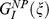
 and 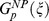 are the values of input and parameter robustness of NP networks (networks that did not pass the Pearson test)
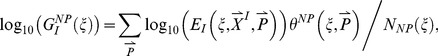
(19)

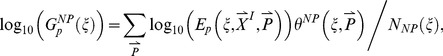
(20)

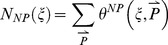
(21)where 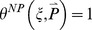 if network 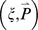 reaches a steady state (see *Selection Criterion* below) but it does not pass the Pearson test and zero otherwise.

We choose the geometric average as more suitable than the arithmetic average as a conservative approach to detecting a possible correlation, as the latter gives too much weight to much larger outliers.

### Selection Criterion

A trial is rejected if it takes too long to reach equilibrium, or its corresponding Jacobian with respect to the node concentrations is singular (i.e., 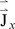 is noninvertible). With this, we obtain 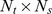 matrices for the relative errors 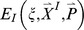 and 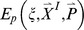 for 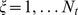 and 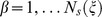. 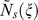 and thus 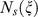 are determined when 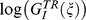, 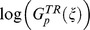, and the fraction of successful trials, 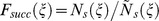, reach equilibrium values. We reject a topology if 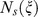 is obviously too small to be statistically significant or 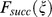 takes too long to reach equilibrium (see below), indicating that the parameter space leading to TR networks for that topology is too small.

We sample over 50,000 different topologies for each 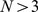 and all possible 3-node topologies (19683), and for each we randomly sample over a large number of parameter sets from a uniform distribution within the ranges 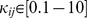 and 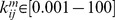 (whenever a link exists between vertices 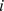 and 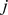). For 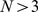 the topologies were randomly generated such that the value of each 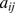 in the corresponding adjacency matrix can take the values −1 or 1 with probability 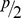 each, and a value 0 with probability 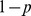. We generated different set of topologies with 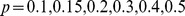. We found no significant difference in the distributions of 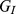 and 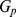 values depending on 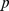. The results shown here represent the collection of all the sets.

We automatically reject trials wherein
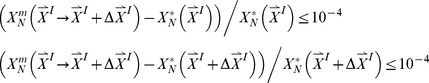
We investigate the effect of the choice of the ranges above by running two separate 3-node simulation. In the first, the parameters are chosen from a uniform distribution in the ranges 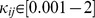 and 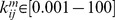, while in the second, the parameters are chosen from a uniform distribution in the ranges 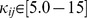 and 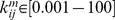. For both ranges we find a significant correlation between robustness to input and parameter perturbations (Fig. S10). For range1 and range2 we obtain the respective values 0.38 and 0.54 for the slopes and 0.73 and 0.68 for the Pearson correlation (Fig. S10A). Furthermore the difference in the slopes becomes insignificant when only networks appearing in both ranges are taken into consideration (Fig. S10B).

### Sensitivity Analysis

As discussed above, the degree of input and parameter robustness is seen as inversely proportional to the average of the sensitivity of the steady state concentration of the output to each input and each parameter, respectively. Then,
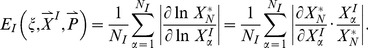
(22)

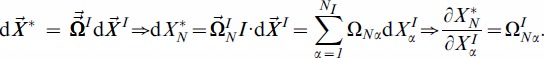
(23)Inserting (22) in (23), we obtain
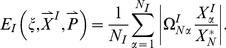
(24)Similarly, for parameter perturbations, 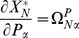 and
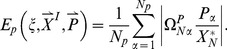
(25)


### Fine-Grained Analysis

In this section we analyze the parameter robustness of different types of parameters. Thus, the 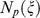 parameters of a topology 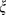 are now divided into 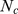 categories. Their corresponding measures of robustness 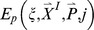 are now defined as
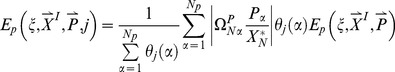
(26)

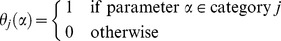
(27)for 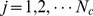.

Thus, the measure of robustness of a topology 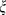 to perturbations in its parameters of category *j* takes the form
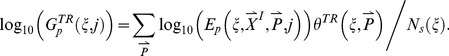
(28)Note that a topology does not have to have parameters belonging to all the defined categories.

Next, to obtain an idea about how robustness to input and parameter perturbations correlate within the networks of each individual topology, we calculate the value 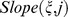 which is the value of the slope obtained from the linear regression on 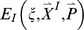 vs 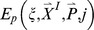 for topology 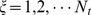 and category 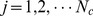.

## Supporting Information

Figure S1 **Distribution of the topologies with different fractions of TR networks, F_succ_**. Linear regression results for 3-node topologies (A): slopes = 0.63, 0.59, 0.53, 0.52, 0.67, and r = 0.67, 0.52, 0.65, 0.67, 0.54 for *F_succ_*<0.8%, *0.8%≤F_succ_*<2.3%, 2.3%≤*F_succ_*<3.3%, 3.3%≤*F_succ_*<4.8%, *F_succ_*≥4.8%, respectively. For 5-node topologies (B): slopes = 0.56, 0.47, 0.54, 0.63, 0.69, and r = 0.52, 0.53, 0.53, 0.45, 0.36 for *F_succ_*<0.8%, *0.8%≤F_succ_*<2.3%, 2.3%≤*F_succ_*<3.3%, 3.3%≤*F_succ_*<4.8%, *F_succ_*≥4.8%, respectively. For 10-node topologies (C): slopes = 0.57, 0.68, 0.72, 0.81, 0.77, and r = 0.35, 0.37, 0.36, 0.34, 0.38 for *F_succ_*<0.8%, *0.8%≤F_succ_*<2.3%, 2.3%≤*F_succ_*<3.3%, 3.3%≤*F_succ_*<4.8%, *F_succ_*≥4.8%, respectively. For 15-node topologies (D): slopes = 0.80, 0.84, 0.86, 0.87, 0.81, and r = 0.21, 0.38, 0.42, 0.39, 0.35 for *F_succ_*<0.8%, *0.8%≤F_succ_*<2.3%, 2.3%≤*F_succ_*<3.3%, 3.3%≤*F_succ_*<4.8%, *F_succ_*≥4.8%, respectively. For 30-node topologies (E): slopes = 1.09, 1.09, 1.12, 1.10, 0.91 and r = 0.37, 0.40, 0.42, 0.42, 0.43 for *F_succ_*<0.8%, *0.8%≤F_succ_*<2.3%, 2.3%≤*F_succ_*<3.3%, 3.3%≤*F_succ_*<4.8%, *F_succ_*≥4.8%, respectively.
(TIF)

Figure S2
**Correlation between robustness to input and parameter perturbations within TR and NP networks before the cutoff.** Correlation between 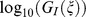 and 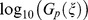 for all topologies showing any number of TR network. 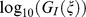 and 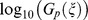 are either computed from the average over TR networks (blue) or from the average over NP networks (green). The linear regression for all sizes (3-node, 5-node, 10-node, 15-node, and 30-node) shows a significant (p<0.0001) correlation between 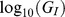 and 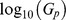. (A) 3-node topologies: Within TR networks, slope = 0.50 (red line: *N* = 4213, r = 0.75). Within NP networks, slope = 0.48 (black line: *N* = 4213, r = 0.60). (B) 5-node topologies: Within TR networks, slope = 0.50 (red line: *N* = 15756, r = 0.56). Within NP networks, slope = 0.39 (black line: *N* = 15756, r = 0.48). (C) 10-node topologies: Within TR networks, slope = 0.66 (red line: *N* = 35522, r = 0.34). Within NP networks, slope = 0.46 (black line: *N* = 35522, r = 0.38). (D) 15-node topologies: Within TR networks, slope = 0.84 (red line: *N* = 39976, r = 0.22). Within NP networks, slope = 0.51 (black line: *N* = 39976, r = 0.36). (E) 30-node topologies: Within TR networks, slope = 0.95 (red line: *N* = 57777, r = 0.35). Within NP networks, slope = 0.54 (black line: *N* = 57301, r = 0.42).(TIF)

Figure S3 **Distribution of E_I_, E_p_ in the original and coarse-grained Ecoli topologies.** (A) Ecoli_1_ is the topology shown in Fig.11A and Ecoli_2_ is its coarse-grained equivalent shown in Fig. 11C. Their corresponding slopes are 0.79 (r = 0.73) and 0.77 (r = 0.86) respectively. (B) Ecoli_1_ is the topology shown in Fig. 11B and Ecoli_2_ is its coarse-grained equivalent shown in Fig. 11D. Their corresponding slopes are −0.98 (r = −0.01, p = 0.85) and 0.37 (r = 0.45, p = 10^−14^) respectively. Here we see more variation in the slope than in (A) as the fraction of TR networks is too low for accurate results.
(TIF)

Figure S4 **Distribution of E_I_, E_p_ for topologies number 1–3, 5–7, 10–12, 16, 19 when the input is a chemo-attractant.** These are the topologies that show no TR networks within the sampled parameter space when the input is a chemo-repellent. The corresponding slopes, r, and P_values are shown in Fig. 12.
(TIF)

Figure S5 **Distribution of E_I_, E_p_ for topologies number 4, 8–9, 13–15, 17–18, 20–27 when the input is a chemo-attractant.** The corresponding slopes, r, and P_values are shown in Fig. 12.
(TIF)

Figure S6 **Distribution of E_I_, E_p_ for topologies number 4, 8–9, 13–15, 17–18, 20–27 when the input is a chemo-repellent.** The corresponding slopes, r, and P_values are shown in Fig. 13.
(TIF)

Figure S7 **Coarse-graining *E. coli* chemotaxis adaptation network.** (A) Our graphical depiction of the original network of E coli Chemotaxis biochemical adaptation as described in [17]–[21]. (B) E coli Chemotaxis adaptation network after redefining the input receiving node. (C) The coarse-grained network.
(TIF)

Figure S8 **Coarse-graining of the Ras model of MAPK cascades.** (A) Our graphical depiction of the original model as described in [53]. (B) Our coarse-grained model.
(TIF)

Figure S9 **Effect of using different criteria for selecting for transiently responsive networks.** Here, four simulations of 3-node topologies are performed using either the Pearson (Pearson1 and Pearson2) or the Spearman (Spearman1 and Spearman2) test. In each case, we use either the definition of *F_ad_* and *F_nad_* in equations (6) and (7) (Pearson1 and Spearman1) or that in equations (10) and (11) (Pearson2 and Spearman2). All simulations resulted in approximately the same results of the slope of the linear regression. (A) compares Pearson1 (red) and Spearman1 (blue). Both resulted in a significant linear correlation (r = 0.72 and r = 0.68, respectively) and no significant difference between the two slopes (0.49 and 0.48, respectively): t_test_ = 0.18 and p = 0.86. Similarly, Pearson2 and Spearman2 (B) resulted in a significant linear correlation (r = 0.60 for both) and no significant difference between the two slopes (0.51 and 0.47, repectively): t_test_ = 1.71 and p = 0.09. Comparing the slopes of Pearson1 and Pearson2 (C), we obtain: t_test_ = 0.86 and p = 0.36. Comparing those of Spearman1 and Spearman2 (D), we obtain: t_test_ = 0.75 and p = 0.46.
(TIF)

Figure S10
**Effect of sampling parameter values from different distributions.** Two simulations are performed where we sample over all 3-node topologies. In both simulations, we sample over parameters sets chosen from a uniform distribution within fixed ranges. In the first (Range1), we set 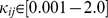 and 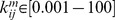 while in the second (Range2), we set 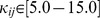 and 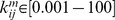. Some topologies only have TR networks within one range but not the other, leading to the different number of topologies in (A). Range1 (red): slope = 0.38 (*N* = 4587, r = 0.73), Range2 (blue): slope = 0.54 (*N* = 3371, r = 0.68), p<0.0001 for both. If only topologies shared between the two are taken into consideration (B), the linear regression shows no significant difference in the slopes (slopes = 0.52 and 0.54 respectively, t_test_ = 0.57, p = 0.57).(TIF)

Figure S11 **Heat maps of the correlations within TR networks.** These are the heat maps corresponding to Fig. 3B (A), Fig. 3C (B), and Fig. 3D (C).
(TIF)

Figure S12 **Heat maps of the correlations within NP networks.** These are the heat maps corresponding to Fig. 4B (A), Fig. 4C (B), and Fig. 4D (C).
(TIF)

Figure S13
**r^2^ within the networks of each 3-node topology divided into 7 categories.** Within each topology 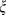, the overall robustness to input perturbations is shown versus 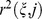 for *j* = 1 (A), 2 (B), 3 (C), 4 (D), 5 (E), 6 (F), and 7 (G).(TIF)

Figure S14
**The slopes within the networks of each 3-node topology divided into 7 categories.** Within each topology 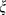, the overall robustness to input perturbations is shown versus 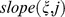 for *j* = 1 (A), 2 (B), 3 (C), 4 (D), 5 (E), 6 (F), and 7 (G).(TIF)

Text S1 S1 Pearson Correlations within TR and NP networks. S2 Steady state analysis.
(DOCX)
