# Supplementary figures and images for: A Network Characteristic That Correlates Environmental and Genetic Robustness
Source: PLoS Comput Biol. 2014 Feb 13;10(2):e1003474. doi: 10.1371/journal.pcbi.1003474 (PMC3923666; doi:10.1371/journal.pcbi.1003474)

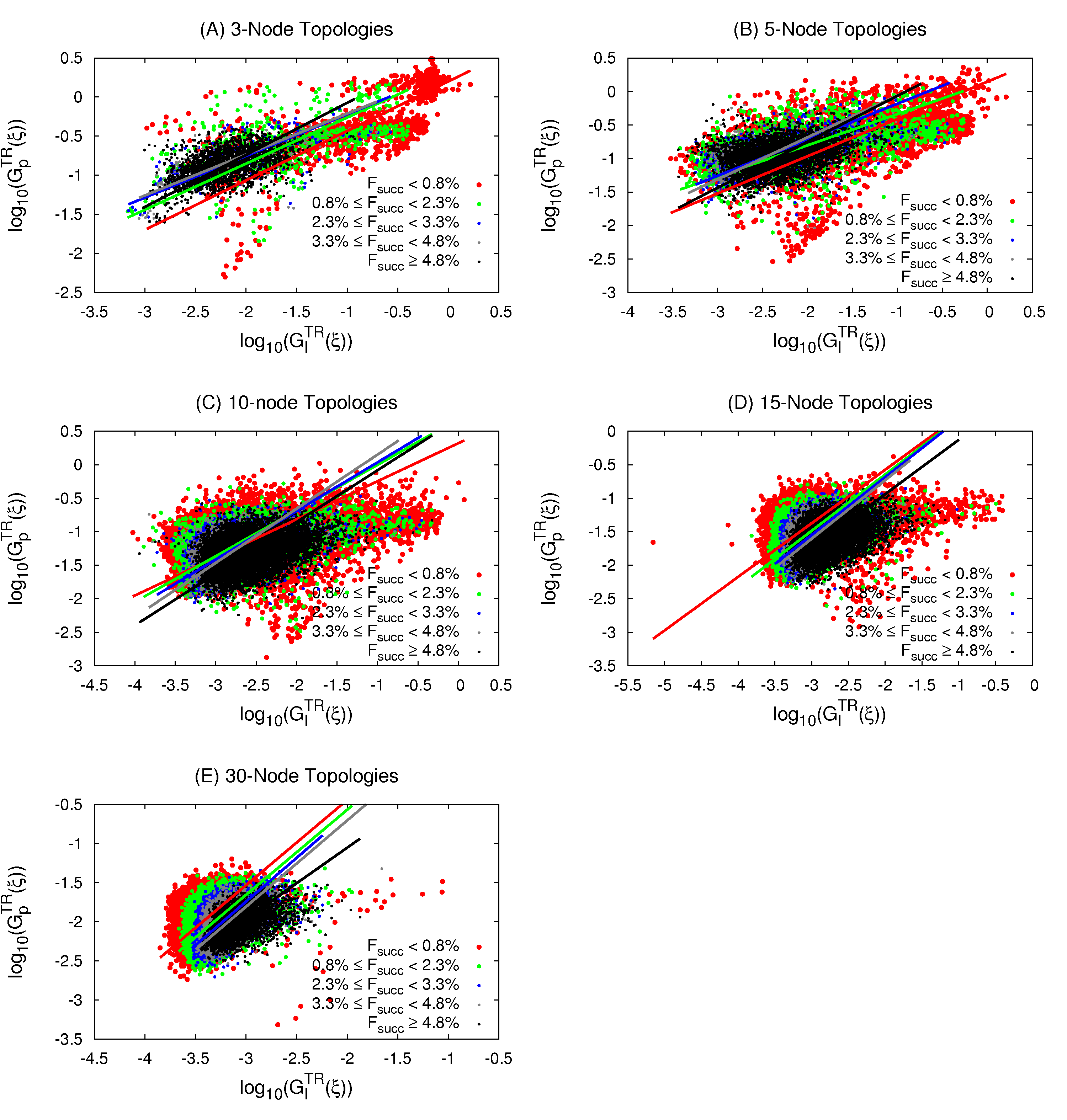

Supplement: Figure S1 — Distribution of the topologies with different fractions of TR networks, Fsucc. Linear regression results for 3-node topologies (A): slopes = 0.63, 0.59, 0.53, 0.52, 0.67, and r = 0.67, 0.52, 0.65, 0.67, 0.54 for Fsucc<0.8%, 0.8%≤Fsucc<2.3%, 2.3%≤Fsucc<3.3%, 3.3%≤Fsucc<4.8%, Fsucc≥4.8%, respectively. For 5-node topologies (B): slopes = 0.56, 0.47, 0.54, 0.63, 0.69, and r = 0.52, 0.53, 0.53, 0.45, 0.36 for Fsucc<0.8%, 0.8%≤Fsucc<2.3%, 2.3%≤Fsucc<3.3%, 3.3%≤Fsucc<4.8%, Fsucc≥4.8%, respectively. For 10-node topologies (C): slopes = 0.57, 0.68, 0.72, 0.81, 0.77, and r = 0.35, 0.37, 0.36, 0.34, 0.38 for Fsucc<0.8%, 0.8%≤Fsucc<2.3%, 2.3%≤Fsucc<3.3%, 3.3%≤Fsucc<4.8%, Fsucc≥4.8%, respectively. For 15-node topologies (D): slopes = 0.80, 0.84, 0.86, 0.87, 0.81, and r = 0.21, 0.38, 0.42, 0.39, 0.35 for Fsucc<0.8%, 0.8%≤Fsucc<2.3%, 2.3%≤Fsucc<3.3%, 3.3%≤Fsucc<4.8%, Fsucc≥4.8%, respectively. For 30-node topologies (E): slopes = 1.09, 1.09, 1.12, 1.10, 0.91 and r = 0.37, 0.40, 0.42, 0.42, 0.43 for Fsucc<0.8%, 0.8%≤Fsucc<2.3%, 2.3%≤Fsucc<3.3%, 3.3%≤Fsucc<4.8%, Fsucc≥4.8%, respectively. (TIF) [file pcbi.1003474.s001.tif]

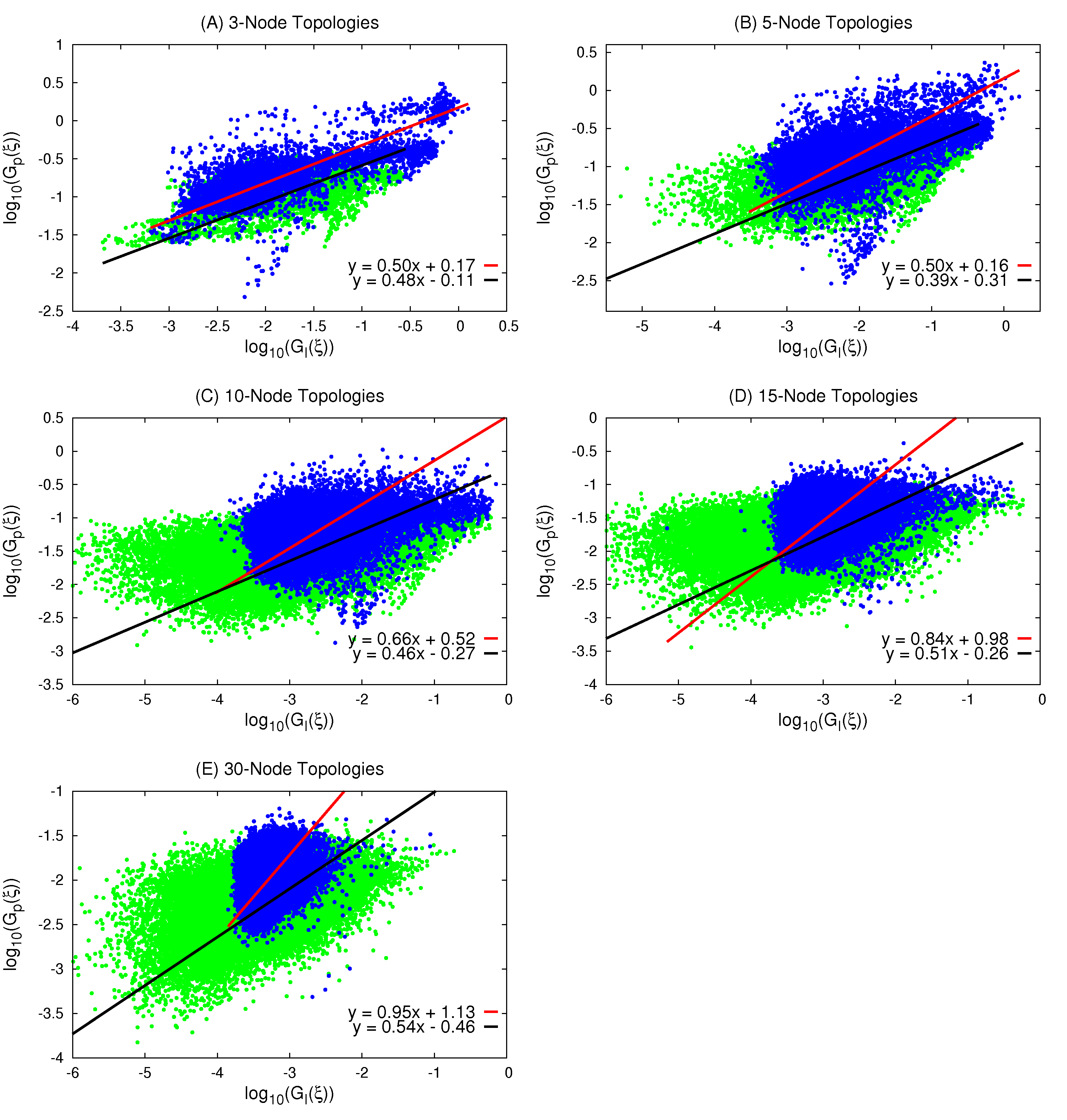

Supplement: Figure S2 — Correlation between robustness to input and parameter perturbations within TR and NP networks before the cutoff. Correlation between and for all topologies showing any number of TR network. and are either computed from the average over TR networks (blue) or from the average over NP networks (green). The linear regression for all sizes (3-node, 5-node, 10-node, 15-node, and 30-node) shows a significant (p<0.0001) correlation between and . (A) 3-node topologies: Within TR networks, slope = 0.50 (red line: N = 4213, r = 0.75). Within NP networks, slope = 0.48 (black line: N = 4213, r = 0.60). (B) 5-node topologies: Within TR networks, slope = 0.50 (red line: N = 15756, r = 0.56). Within NP networks, slope = 0.39 (black line: N = 15756, r = 0.48). (C) 10-node topologies: Within TR networks, slope = 0.66 (red line: N = 35522, r = 0.34). Within NP networks, slope = 0.46 (black line: N = 35522, r = 0.38). (D) 15-node topologies: Within TR networks, slope = 0.84 (red line: N = 39976, r = 0.22). Within NP networks, slope = 0.51 (black line: N = 39976, r = 0.36). (E) 30-node topologies: Within TR networks, slope = 0.95 (red line: N = 57777, r = 0.35). Within NP networks, slope = 0.54 (black line: N = 57301, r = 0.42). (TIF) [file pcbi.1003474.s002.tif]

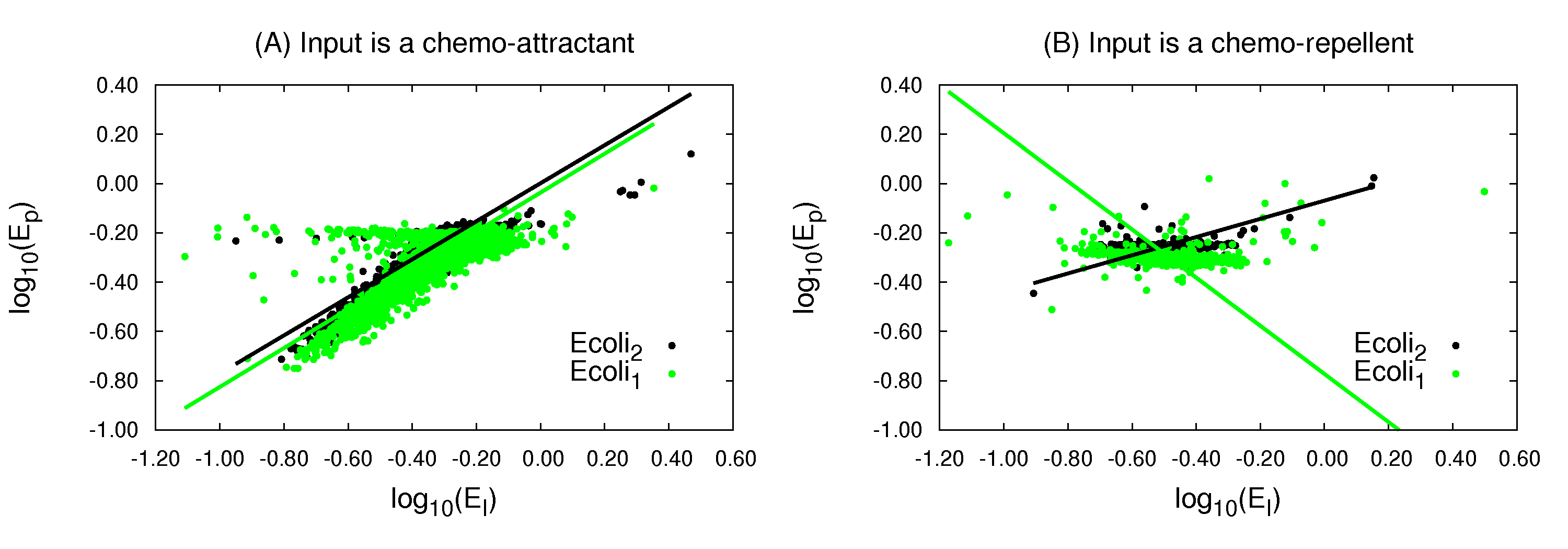

Supplement: Figure S3 — Distribution of EI, Ep in the original and coarse-grained Ecoli topologies. (A) Ecoli1 is the topology shown in Fig.11A and Ecoli2 is its coarse-grained equivalent shown in Fig. 11C. Their corresponding slopes are 0.79 (r = 0.73) and 0.77 (r = 0.86) respectively. (B) Ecoli1 is the topology shown in Fig. 11B and Ecoli2 is its coarse-grained equivalent shown in Fig. 11D. Their corresponding slopes are −0.98 (r = −0.01, p = 0.85) and 0.37 (r = 0.45, p = 10−14) respectively. Here we see more variation in the slope than in (A) as the fraction of TR networks is too low for accurate results. (TIF) [file pcbi.1003474.s003.tif]

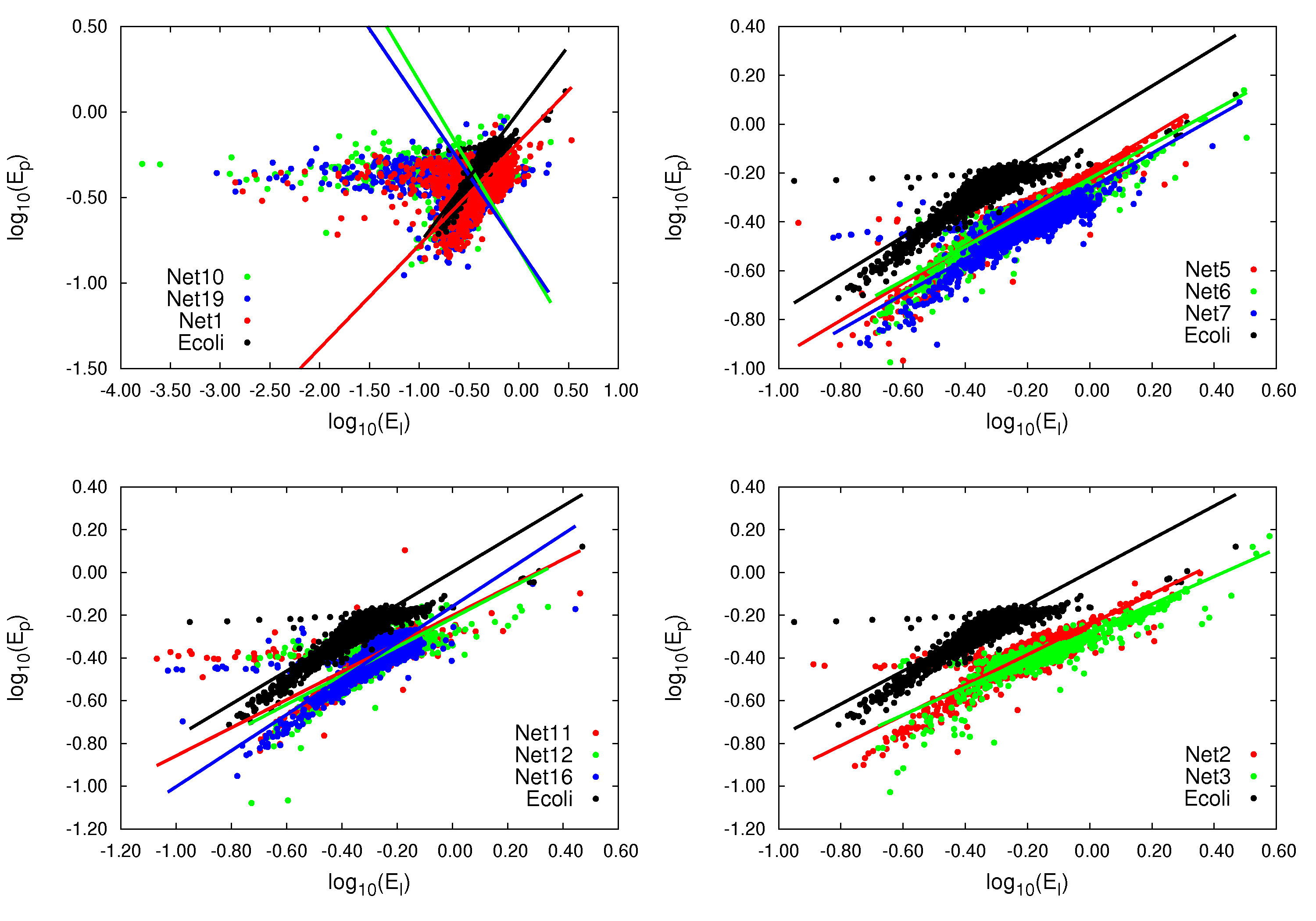

Supplement: Figure S4 — Distribution of EI, Ep for topologies number 1–3, 5–7, 10–12, 16, 19 when the input is a chemo-attractant. These are the topologies that show no TR networks within the sampled parameter space when the input is a chemo-repellent. The corresponding slopes, r, and P_values are shown in Fig. 12. (TIF) [file pcbi.1003474.s004.tif]

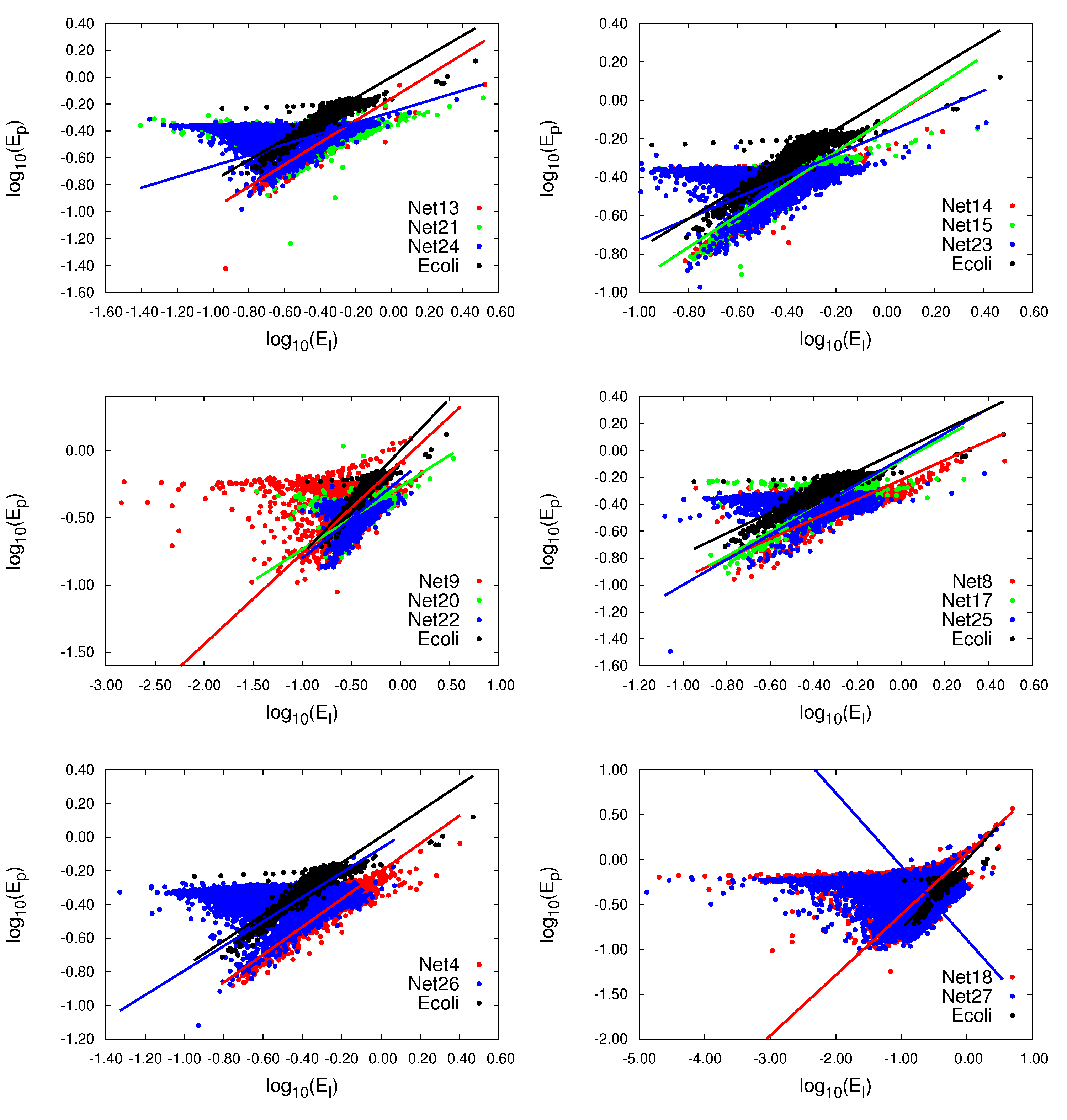

Supplement: Figure S5 — Distribution of EI, Ep for topologies number 4, 8–9, 13–15, 17–18, 20–27 when the input is a chemo-attractant. The corresponding slopes, r, and P_values are shown in Fig. 12. (TIF) [file pcbi.1003474.s005.tif]

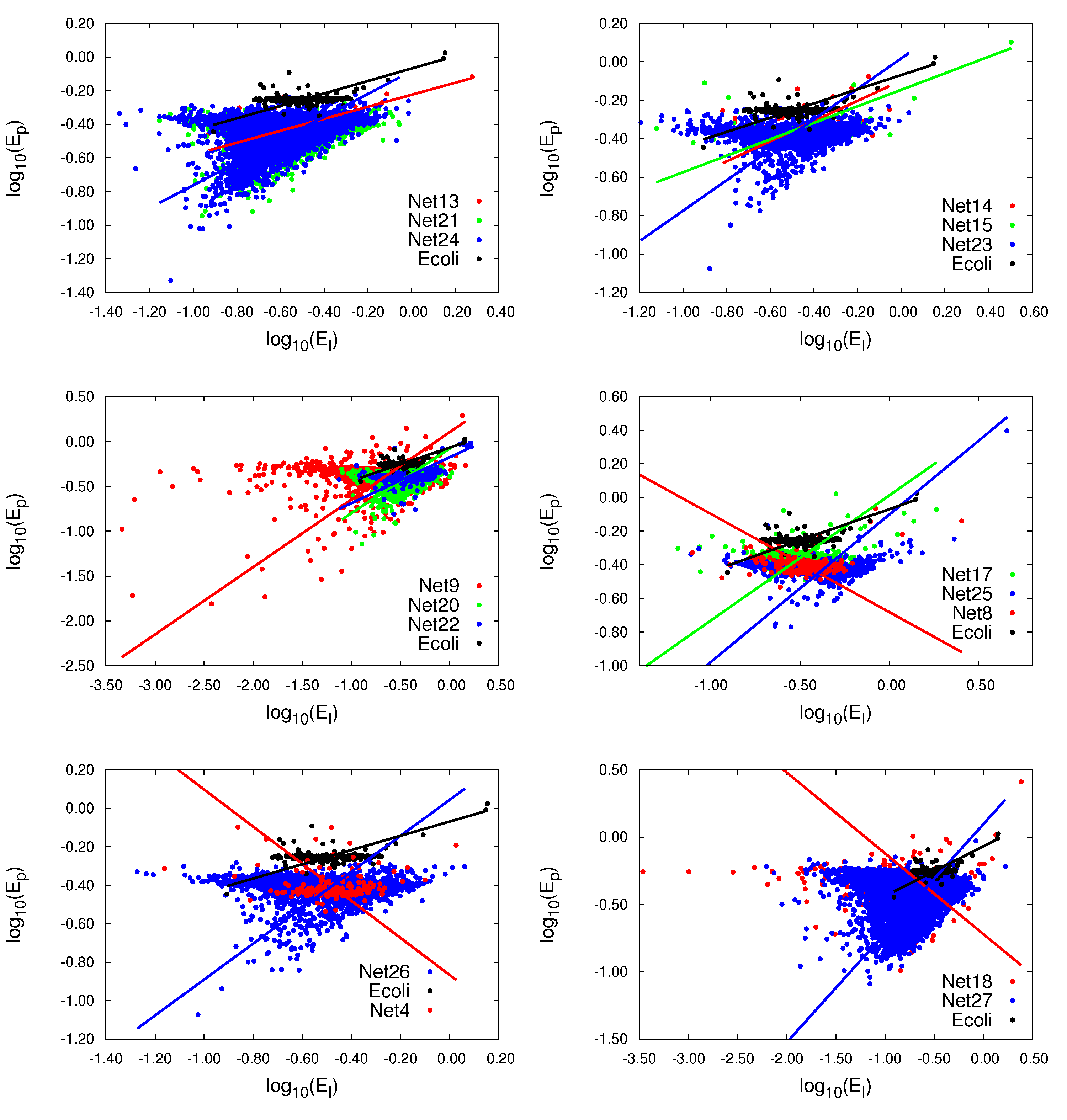

Supplement: Figure S6 — Distribution of EI, Ep for topologies number 4, 8–9, 13–15, 17–18, 20–27 when the input is a chemo-repellent. The corresponding slopes, r, and P_values are shown in Fig. 13. (TIF) [file pcbi.1003474.s006.tif]

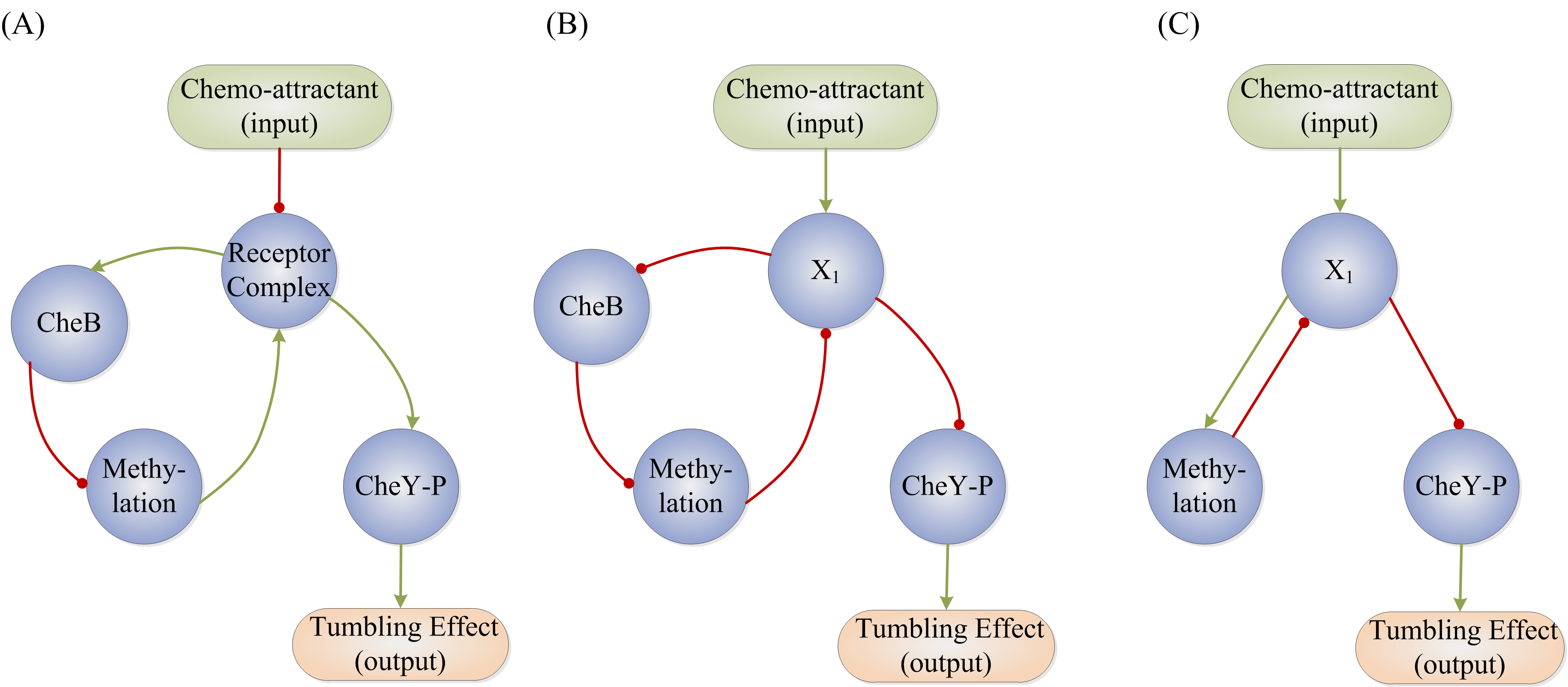

Supplement: Figure S7 — Coarse-graining E. coli chemotaxis adaptation network. (A) Our graphical depiction of the original network of E coli Chemotaxis biochemical adaptation as described in [17]–[21]. (B) E coli Chemotaxis adaptation network after redefining the input receiving node. (C) The coarse-grained network. (TIF) [file pcbi.1003474.s007.tif]

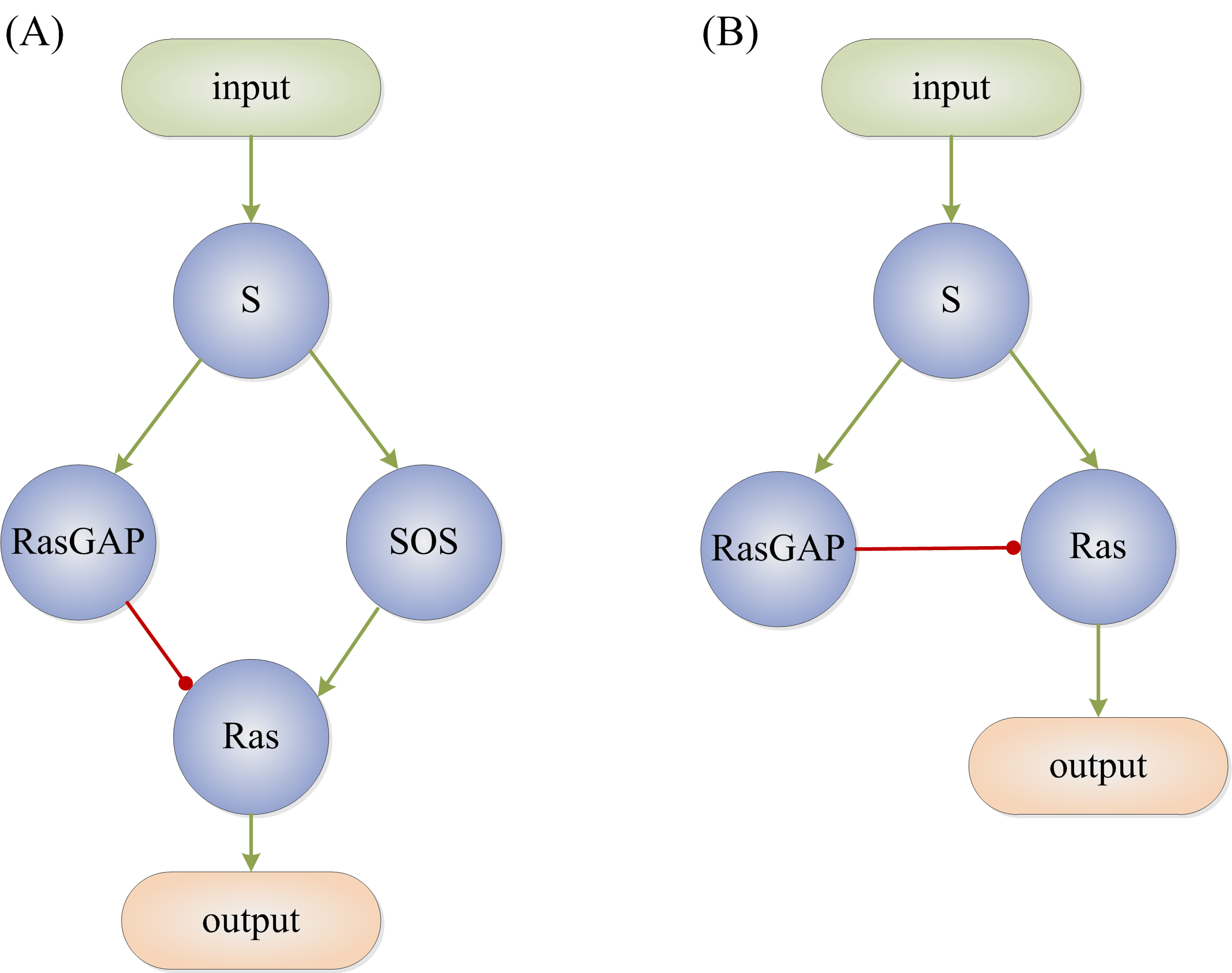

Supplement: Figure S8 — Coarse-graining of the Ras model of MAPK cascades. (A) Our graphical depiction of the original model as described in [53]. (B) Our coarse-grained model. (TIF) [file pcbi.1003474.s008.tif]

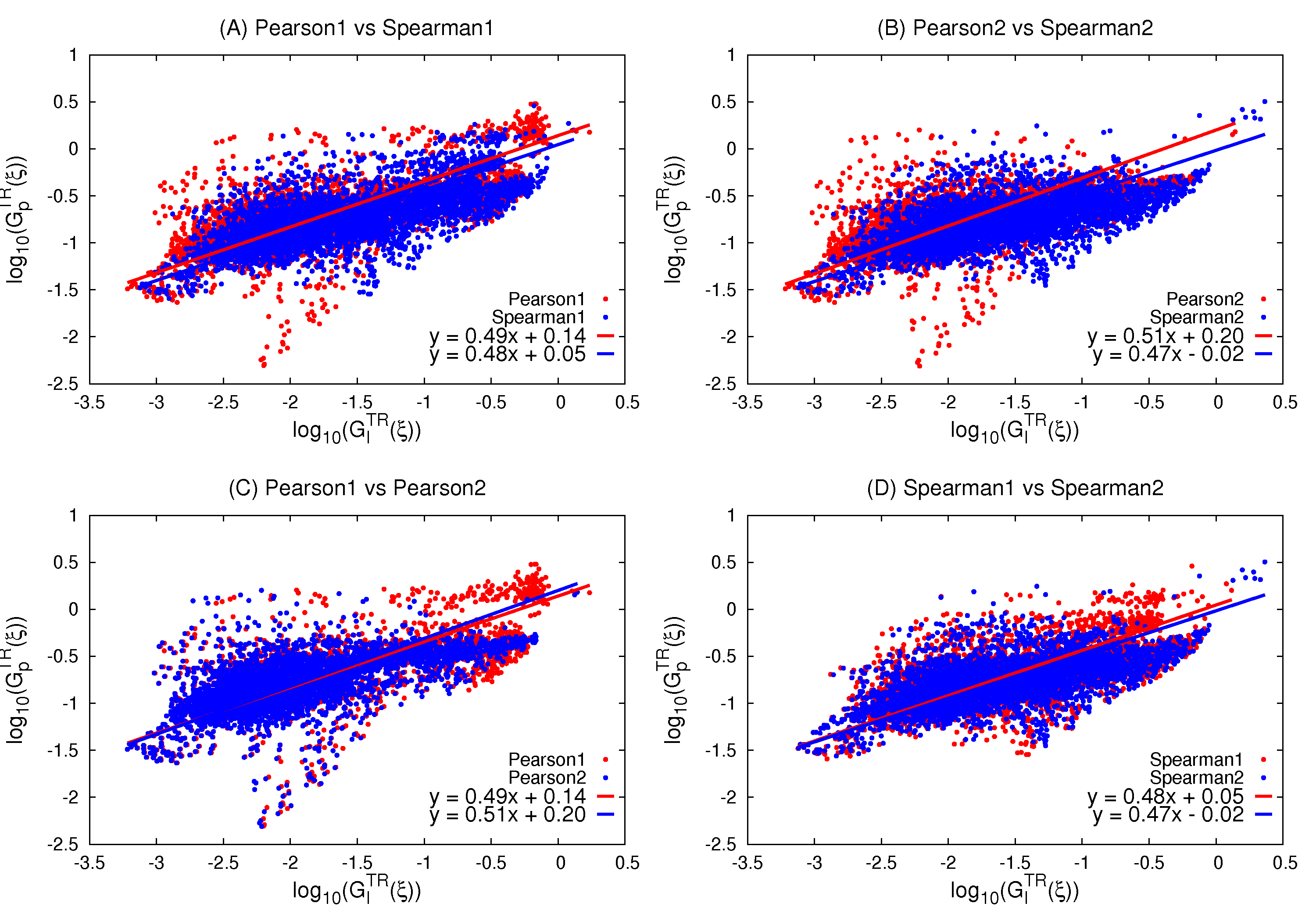

Supplement: Figure S9 — Effect of using different criteria for selecting for transiently responsive networks. Here, four simulations of 3-node topologies are performed using either the Pearson (Pearson1 and Pearson2) or the Spearman (Spearman1 and Spearman2) test. In each case, we use either the definition of Fad and Fnad in equations (6) and (7) (Pearson1 and Spearman1) or that in equations (10) and (11) (Pearson2 and Spearman2). All simulations resulted in approximately the same results of the slope of the linear regression. (A) compares Pearson1 (red) and Spearman1 (blue). Both resulted in a significant linear correlation (r = 0.72 and r = 0.68, respectively) and no significant difference between the two slopes (0.49 and 0.48, respectively): ttest = 0.18 and p = 0.86. Similarly, Pearson2 and Spearman2 (B) resulted in a significant linear correlation (r = 0.60 for both) and no significant difference between the two slopes (0.51 and 0.47, repectively): ttest = 1.71 and p = 0.09. Comparing the slopes of Pearson1 and Pearson2 (C), we obtain: ttest = 0.86 and p = 0.36. Comparing those of Spearman1 and Spearman2 (D), we obtain: ttest = 0.75 and p = 0.46. (TIF) [file pcbi.1003474.s009.tif]

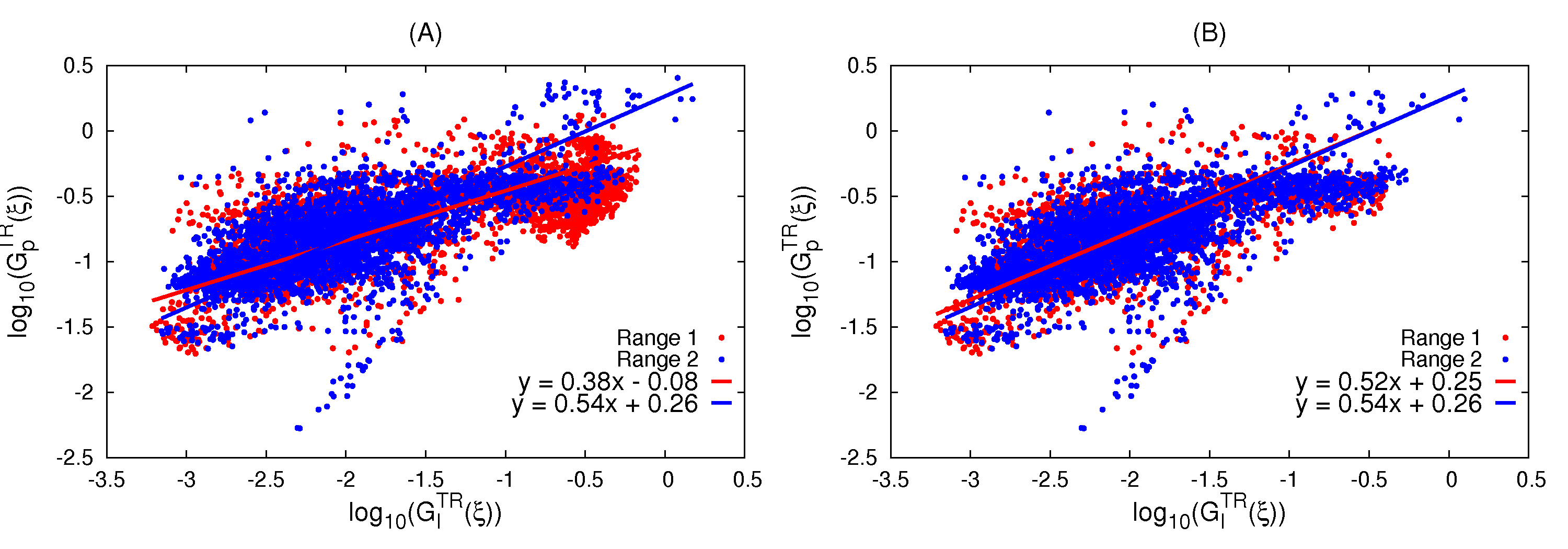

Supplement: Figure S10 — Effect of sampling parameter values from different distributions. Two simulations are performed where we sample over all 3-node topologies. In both simulations, we sample over parameters sets chosen from a uniform distribution within fixed ranges. In the first (Range1), we set and while in the second (Range2), we set and . Some topologies only have TR networks within one range but not the other, leading to the different number of topologies in (A). Range1 (red): slope = 0.38 (N = 4587, r = 0.73), Range2 (blue): slope = 0.54 (N = 3371, r = 0.68), p<0.0001 for both. If only topologies shared between the two are taken into consideration (B), the linear regression shows no significant difference in the slopes (slopes = 0.52 and 0.54 respectively, ttest = 0.57, p = 0.57). (TIF) [file pcbi.1003474.s010.tif]

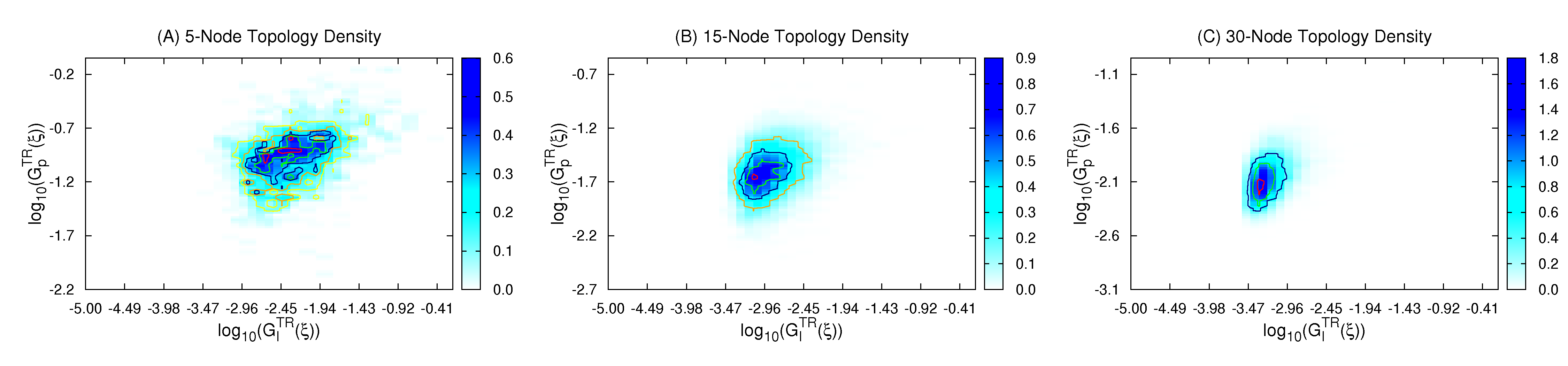

Supplement: Figure S11 — Heat maps of the correlations within TR networks. These are the heat maps corresponding to Fig. 3B (A), Fig. 3C (B), and Fig. 3D (C). (TIF) [file pcbi.1003474.s011.tif]

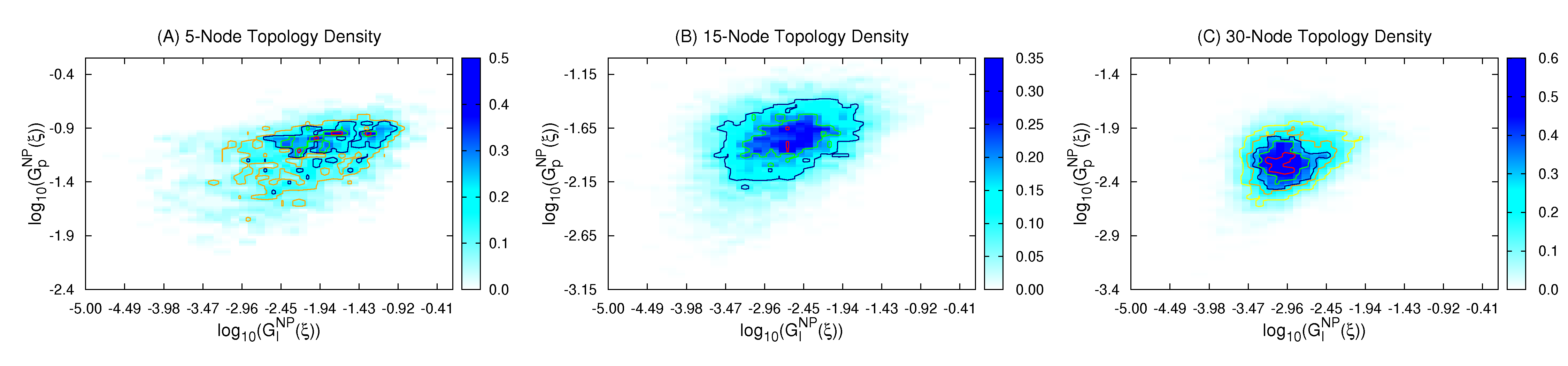

Supplement: Figure S12 — Heat maps of the correlations within NP networks. These are the heat maps corresponding to Fig. 4B (A), Fig. 4C (B), and Fig. 4D (C). (TIF) [file pcbi.1003474.s012.tif]

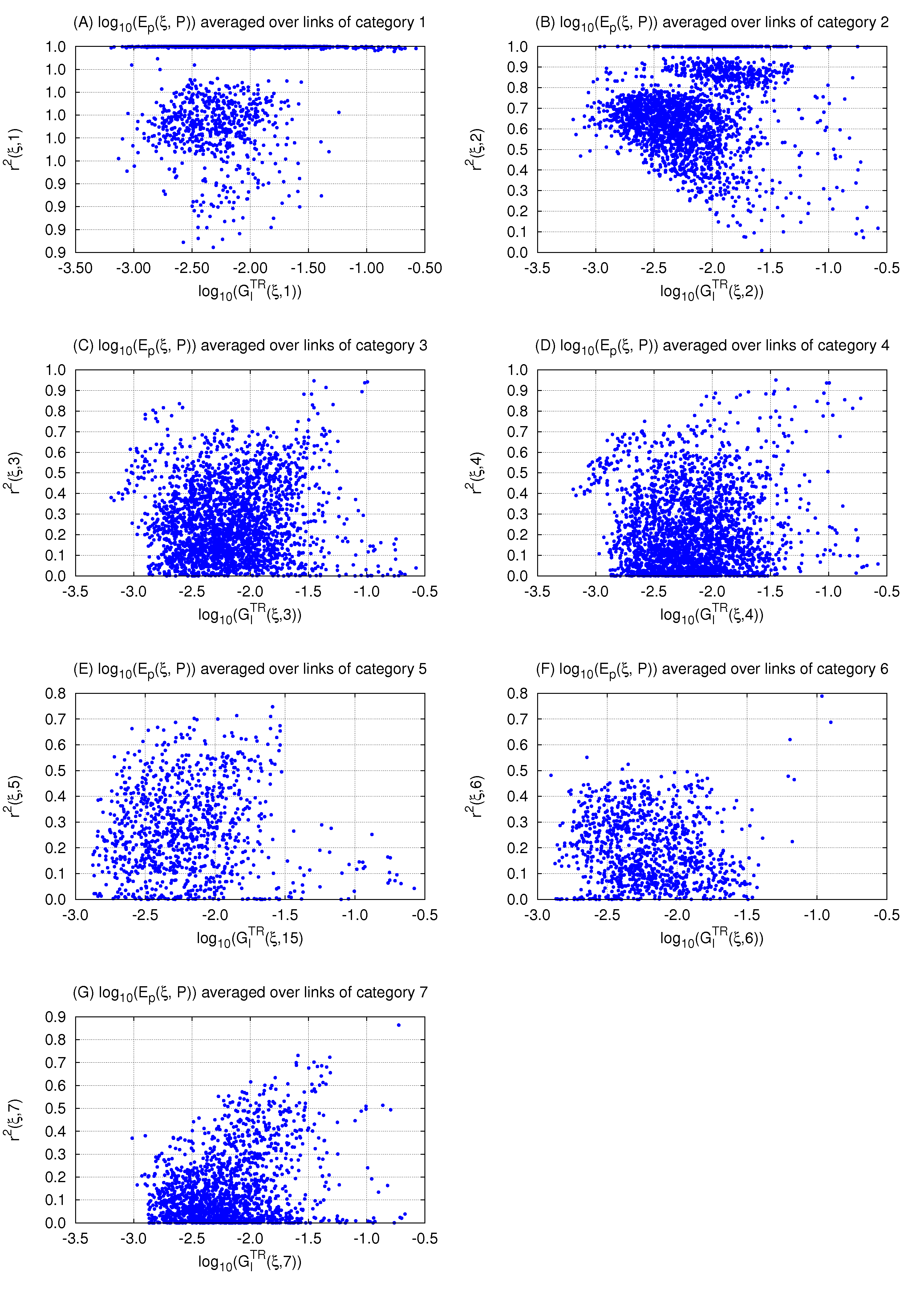

Supplement: Figure S13 — r2 within the networks of each 3-node topology divided into 7 categories. Within each topology , the overall robustness to input perturbations is shown versus for j = 1 (A), 2 (B), 3 (C), 4 (D), 5 (E), 6 (F), and 7 (G). (TIF) [file pcbi.1003474.s013.tif]

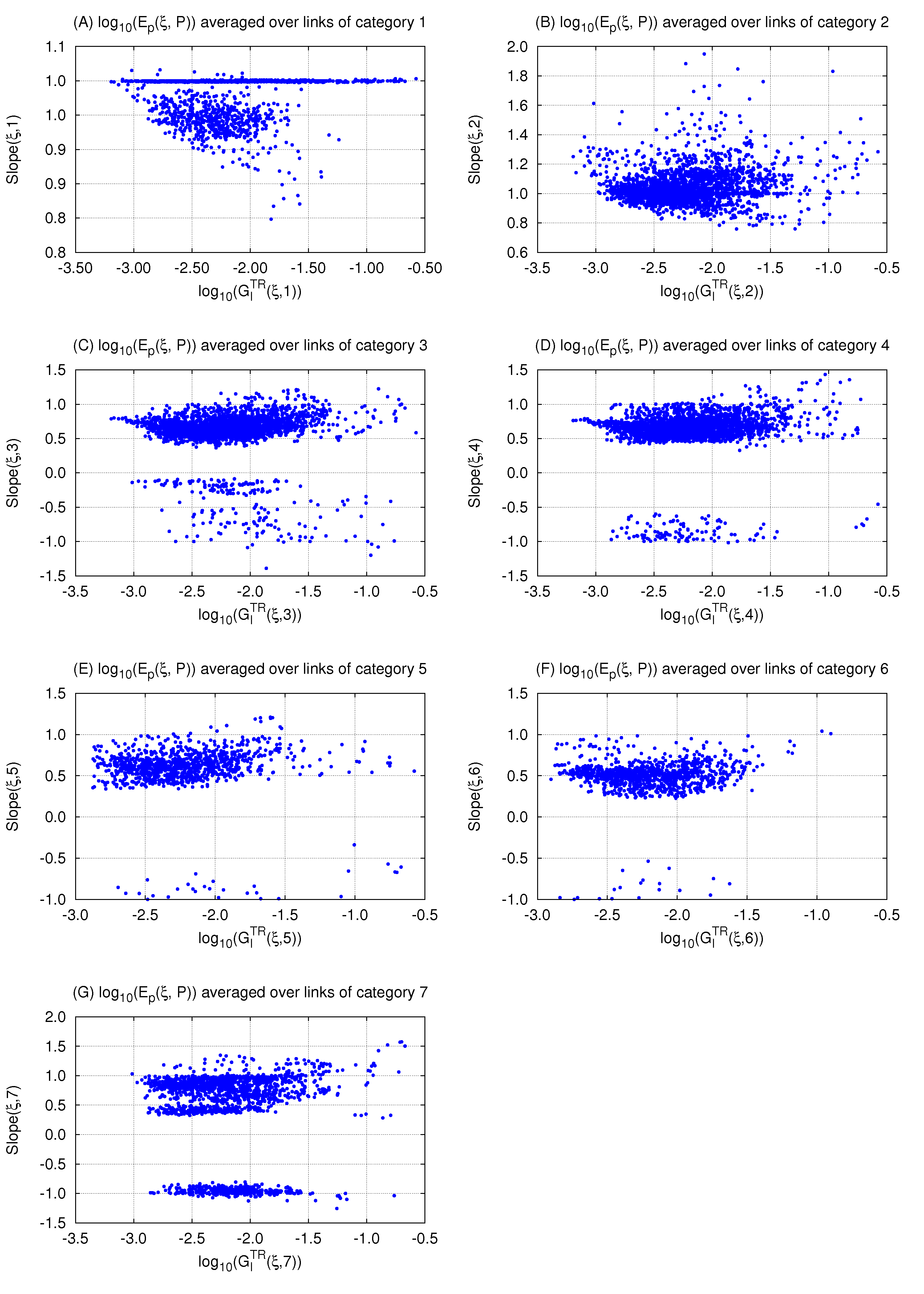

Supplement: Figure S14 — The slopes within the networks of each 3-node topology divided into 7 categories. Within each topology , the overall robustness to input perturbations is shown versus for j = 1 (A), 2 (B), 3 (C), 4 (D), 5 (E), 6 (F), and 7 (G). (TIF) [file pcbi.1003474.s014.tif]
