# Supplementary material for: A Network Characteristic That Correlates Environmental and Genetic Robustness
Source: PLoS Comput Biol. 2014 Feb 13;10(2):e1003474. doi: 10.1371/journal.pcbi.1003474 (PMC3923666; doi:10.1371/journal.pcbi.1003474)
Supplement: Text S1 — S1 Pearson Correlations within TR and NP networks. S2 Steady state analysis. (DOCX) [file pcbi.1003474.s015.docx]

**S1 Pearson Correlations Within TR and NP Networks**

Within TR networks, the Pearson correlation, r, decreases as the size of the networks increases from r = 0.57 for 3-node to r = 0.35 for 15-node topologies, but start increasing again for 30-node topologies with r = 0.41. Within NP networks, the Pearson correlation consistently decreases with the size of the networks from r = 0.61 for 3-node to r = 0.30 for 30-node topologies. Similarly, while r is slightly higher within NP networks than within TR networks of the same size for 3-node, 5-node, and 15-node, it is lower for 30-node topologies (r = 0.30 for NP versus 0.41 for TR). Furthermore, the scatter decreases as the size of the networks increases for TR networks (Fig. S11), but not as much for NP networks except for the 30-node networks (Fig. S12). The reason r initially decreases with the size of the networks is due to the increase in the number of parameters whose robustness stay invariant and possibly do not contribute to the robustness to input perturbations either (see Fig. 8 and the discussion in the *Fine-Grained Analysis Within 3-node Topologies* section below). The Pearson correlation within TR networks is skewed due to the additional scattering; note how r is higher for 3-node and 5-node TR networks before the cutoff (Fig. S2, A-B) than after the cutoff (Fig. 3A-B). Also, TR networks include those that are barely responsive to input changes as indicated by the much smaller $\log_{10}\left(G_{I}^{NP}\right)$ included (Fig. S2).

**S2 Steady State Analysis**

We derive equations to quantify the change in output due to change in input concentration and parameters. First, we rewrite the rate functions of equation (3) as responsive to the varying enzymes, $\vec{X}$, input, $\vec{X}^{I}$, and parameters, $\vec{P}$.

$\vec{F}\left(t\right)\equiv\vec{F}\left[\vec{X}\left(t\right),\vec{X}^{I},\vec{P};t\right]$, (S1)

where the dependency on basal enzymes is dropped from the notation for simplicity, as they are constant and are never perturbed.

At steady state,

$\vec{F}\left[\vec{X}^{*},\vec{X}^{I},\vec{P}\right]=0$ (S2)

where $\vec{X}^{*}\left(\vec{X}^{I},\vec{P}\right)$ is the set of steady state concentrations for input $\vec{X}^{I}$ and parameter set $\vec{P}$.

***Perturbation in Input***

Expanding $\vec{F}\left[\vec{X}\left(t\right),\vec{X}^{I}+d\vec{X}^{I},\vec{P};t\right]$ around $\vec{X}^{*}\left(\vec{X}^{I},\vec{P}\right)$ and $\vec{X}^{I}$ yields

(S3)

At steady state, we obtain

(S4)

$$\Rightarrow\left[\begin{matrix} \frac{\partial F_{1}}{\partial X_{1}} & \frac{\partial F_{1}}{\partial X_{2}} & \cdots& \frac{\partial F_{1}}{\partial X_{N}} \\ \frac{\partial F_{2}}{\partial X_{1}} & \frac{\partial F_{2}}{\partial X_{2}} & \cdots& \frac{\partial F_{2}}{\partial X_{N}} \\ \vdots& \vdots& \vdots& \vdots\\ \frac{\partial F_{N}}{\partial X_{1}} & \frac{\partial F_{N}}{\partial X_{2}} & \cdots& \frac{\partial F_{N}}{\partial X_{N}} \end{matrix}\right]\left[\begin{matrix} dX_{1}^{*} \\ dX_{2}^{*} \\ \vdots\\ dX_{N}^{*} \end{matrix}\right]=-\left[\begin{matrix} \frac{\partial F_{1}}{\partial X_{1}^{I}} & \frac{\partial F_{1}}{\partial X_{2}^{I}} & \cdots& \frac{\partial F_{1}}{\partial X_{N_{I}}^{I}} \\ \frac{\partial F_{2}}{\partial X_{1}^{I}} & \frac{\partial F_{2}}{\partial X_{2}^{I}} & \cdots& \frac{\partial F_{2}}{\partial X_{N_{I}}^{I}} \\ \vdots& \vdots& \vdots& \vdots\\ \frac{\partial F_{N}}{\partial X_{1}^{I}} & \frac{\partial F_{N}}{\partial X_{2}^{I}} & \cdots& \frac{\partial F_{N}}{\partial X_{N_{I}}^{I}} \end{matrix}\right]\left[\begin{matrix} dX_{1}^{I} \\ dX_{2}^{I} \\ \vdots\\ dX_{N}^{I} \end{matrix}\right]\Rightarrow\vec{\vec{J}}_{x}d\vec{X}^{*}=-\vec{\vec{J}}_{I}d\vec{X}^{I}$$

where $\vec{\vec{J}}_{x}$ and $\vec{\vec{J}}_{I}$ are the $N\times N$ and $N\times N_{I}$ Jacobian matrices with respect to the node and input concentrations respectively. Therefore

$d\vec{X}^{*}=-\vec{\vec{J}}_{x}^{-1}\vec{\vec{J}}_{I}d\vec{X}^{I}\equiv\vec{\vec{\Omega}}^{I}d\vec{X}^{I}$ (S5)

***Perturbation in Parameters***

As above, we seek an explicit expression for $\Delta\tilde{X}_{N}^{*}$ so that the computational task is feasible for large samples of networks. We start by expanding $\vec{F}\left[\vec{X}\left(t\right),\vec{X}^{I},\vec{P}+d\vec{P};t\right]$ around $\vec{X}^{*}\left(\vec{X}^{I},\vec{P}\right)$ and $\vec{P}$

(S6)

At steady state, we obtain

(S7)

$$\Rightarrow\left[\begin{matrix} \frac{\partial F_{1}}{\partial X_{1}} & \frac{\partial F_{1}}{\partial X_{2}} & \cdots& \frac{\partial F_{1}}{\partial X_{N}} \\ \frac{\partial F_{2}}{\partial X_{1}} & \frac{\partial F_{2}}{\partial X_{2}} & \cdots& \frac{\partial F_{2}}{\partial X_{N}} \\ \vdots& \vdots& \vdots& \vdots\\ \frac{\partial F_{N}}{\partial X_{1}} & \frac{\partial F_{N}}{\partial X_{2}} & \cdots& \frac{\partial F_{N}}{\partial X_{N}} \end{matrix}\right]\left[\begin{matrix} dX_{1}^{*} \\ dX_{2}^{*} \\ \vdots\\ dX_{N}^{*} \end{matrix}\right]=-\left[\begin{matrix} \frac{\partial F_{1}}{\partial P_{1}} & \frac{\partial F_{1}}{\partial P_{2}} & \cdots& \frac{\partial F_{1}}{\partial P_{N_{p}}} \\ \frac{\partial F_{2}}{\partial P_{1}} & \frac{\partial F_{2}}{\partial P_{2}} & \cdots& \frac{\partial F_{2}}{\partial P_{N_{p}}} \\ \vdots& \vdots& \vdots& \vdots\\ \frac{\partial F_{N}}{\partial P_{1}} & \frac{\partial F_{N}}{\partial P_{2}} & \cdots& \frac{\partial F_{N}}{\partial P_{N_{p}}} \end{matrix}\right]\left[\begin{matrix} dP_{1} \\ dP_{2} \\ \vdots\\ dP_{N_{p}} \end{matrix}\right]\Rightarrow\vec{\vec{J}}_{x}d\vec{X}^{*}=-\vec{\vec{J}}_{p}d\vec{P}$$

where $\vec{\vec{J}}_{p}$ is the $N\times N_{p}$ Jacobian matrix with respect to the parameters. Therefore,

$d\vec{X}^{*}=-\vec{\vec{J}}_{x}^{-1}\vec{\vec{J}}_{p}d\vec{P}\equiv\vec{\vec{\Omega}}^{P}d\vec{P}$ (S8)
